# PEGASUS: Prediction of MD‐derived protein flexibility from sequence

**DOI:** 10.1002/pro.70221

**Published:** 2025-07-16

**Authors:** Yann Vander Meersche, Gabriel Duval, Gabriel Cretin, Aria Gheeraert, Jean‐Christophe Gelly, Tatiana Galochkina

**Affiliations:** ^1^ Université Paris Cité and Université des Antilles and Université de la Réunion, INSERM, BIGR, DSIMB Paris France

**Keywords:** deep learning, molecular dynamics, prediction of protein properties, protein dynamics, protein flexibility, protein language models, sequence‐based predictions

## Abstract

Protein flexibility is essential to its biological function. However, experimental methods for its assessment, such as X‐ray crystallography and nuclear magnetic resonance spectroscopy, are often limited by experimental variability and high cost, leading to a gap between the number of identified protein sequences and the available experimental information on protein dynamics. On the other hand, molecular dynamics (MD) simulations provide a uniform and detailed description of the expected protein flexibility, and the availability and quality of such data are increasing significantly during the last years. In this study, we use the recently released ATLAS database to develop ProtEin lanGuAge models for prediction of SimUlated dynamicS (PEGASUS), a sequence‐based predictor of MD‐derived information on protein flexibility (https://dsimb.inserm.fr/PEGASUS). PEGASUS integrates four different representations of protein sequences generated by Protein Language Models to predict residue‐wise MD‐derived values of backbone fluctuation (root mean square fluctuation), Phi and Psi dihedral angles standard deviation, and average Local Distance Difference Test across the trajectory. The PEGASUS web server was optimized to perform instantaneous predictions for an individual protein sequence and also allows batch submission of up to 100 sequences of 1 k residues each. For more complex queries, we also release PEGASUS as a user‐friendly standalone utility (https://github.com/DSIMB/PEGASUS).

## 1 INTRODUCTION

Protein function is largely determined by its molecular structure and associated dynamic properties. Proteins constantly undergo structural changes of varying amplitude and frequency. Therefore, the flexibility of different protein regions determines its molecular function in various biological mechanisms (Teilum et al., 2009). However, available experimental information on protein dynamics, such as B‐factor values obtained by X‐ray crystallography and the general order parameter *S* ^2^ and conformational ensembles from nuclear magnetic resonance (NMR) spectroscopy, remains limited and highly dependent on the experimental conditions (Carugo, 2018, 2022; Sun et al., 2019).

Due to the recent breakthrough in protein structure prediction (Jumper et al., 2021), reliable static three‐dimensional coordinates of protein atoms become available for a vast majority of globular protein sequences. At the same time, tools for predicting the accompanying dynamics properties remain underdeveloped. Most of the developed tools aim to predict protein flexibility in terms of B‐factor, since it is the most abundant experimental flexibility measurement of the protein data bcank (PDB) with over 166 k X‐ray structures compared to 12 k NMR ensembles available as of September 2024. The most recent approaches include PROFBval (Schlessinger et al., 2006), PredyFlexy (de Brevern et al., 2012), FlexC (Yaseen et al., 2016), and MEDUSA (Vander Meersche et al., 2021). All the mentioned tools discretize B‐factor values into different flexibility categories in order to overcome inherent issues with B‐factor measurements influenced by experimental conditions (Carugo, 2018, 2022; Sun et al., 2019). Nevertheless, the heterogeneity of B‐factor values even for crystals of the same protein (Carugo, 2022) and the absence of information on flexible protein regions such as loops and protein termini significantly limit the performance of the tools mentioned above, estimated to be 83% for binary predictions (Schlessinger et al., 2006; Schlessinger & Rost, 2005). Other prediction methods, such as DynaMine (Cilia et al., 2013) based on NMR chemical shift information, encounter similar limitations, such as the lack of training data availability for large proteins as well as the heterogeneity of experimental conditions.

Molecular dynamics (MD) simulations offer an alternative way to assess local and global protein flexibility through the analysis of protein motion simulated over nanosecond to microsecond timescales (Collier et al., 2020). For today, PredyFlexy (de Brevern et al., 2012) published in 2012 remains the only tool that uses MD‐derived metrics to predict protein flexibility directly from sequence. It categorizes flexibility into three classes (rigid, intermediate, and flexible) by combining B‐factors with root mean square fluctuation (RMSF) data from MD simulations. A decade after the release of PredyFlexy, we observe the emergence of several initiatives focused on MD simulation data generation, storage, and sharing (Liu et al., 2024; Mirarchi et al., 2024; Vander Meersche et al., 2024). As a result, the community can benefit from access to much more abundant MD data of higher quality, such as ATLAS, a comprehensive dataset of all‐atom MD simulations of more than 1 k proteins representative of the existing protein folds developed by our group (Vander Meersche et al., 2024).

This study introduces ProtEin lanGuAge models for prediction of SimUlated dynamicS (PEGASUS), a sequence‐based protein flexibility predictor trained on MD‐derived metrics computed for all proteins of the ATLAS database. PEGASUS leverages protein Language Models (pLMs) (Elnaggar et al., 2022; Lin et al., 2023) and deep learning techniques to accurately predict key flexibility metrics, such as RMSF, dihedral angle standard deviations, and Average Local Distance Difference Test (Mean LDDT) values. It outperforms existing methods like PredyFlexy and MEDUSA on similar tasks, and shows strong correlations with MD simulation data. Moreover, PEGASUS demonstrates good performance when compared with experimental disorder measurements and AlphaFold 2's predicted Local Distance Difference Test (pLDDT). Additionally, it effectively captures both local and global impacts of mutations, accurately identifying stabilizing and destabilizing effects in proteins such as p53, xylanase, and bovine pancreatic trypsin inhibitor (BPTI). Finally, PEGASUS is accessible as a free web server (https://dsimb.inserm.fr/PEGASUS/) allowing instantaneous predictions for individual proteins and advanced options such as batch submission and comparison of predictions obtained for aligned protein sequences. PEGASUS is also available as a standalone Docker image (https://github.com/DSIMB/pegasus) for advanced usage.

## 2 RESULTS

To compare PEGASUS performance against other existing methods, we run predictions on an independent test set consisting of 15 CASP15 (Critical Assessment of Techniques for Protein Structure Prediction) free‐modeling targets. For each protein of the test dataset, we generated MD trajectories following the same protocol as used in the ATLAS database and calculated reference RMSF profiles for the resulting 100 ns trajectories.

### 2.1 PEGASUS outperforms state‐of‐the‐art tools for protein flexibility prediction

**FIGURE 1:**
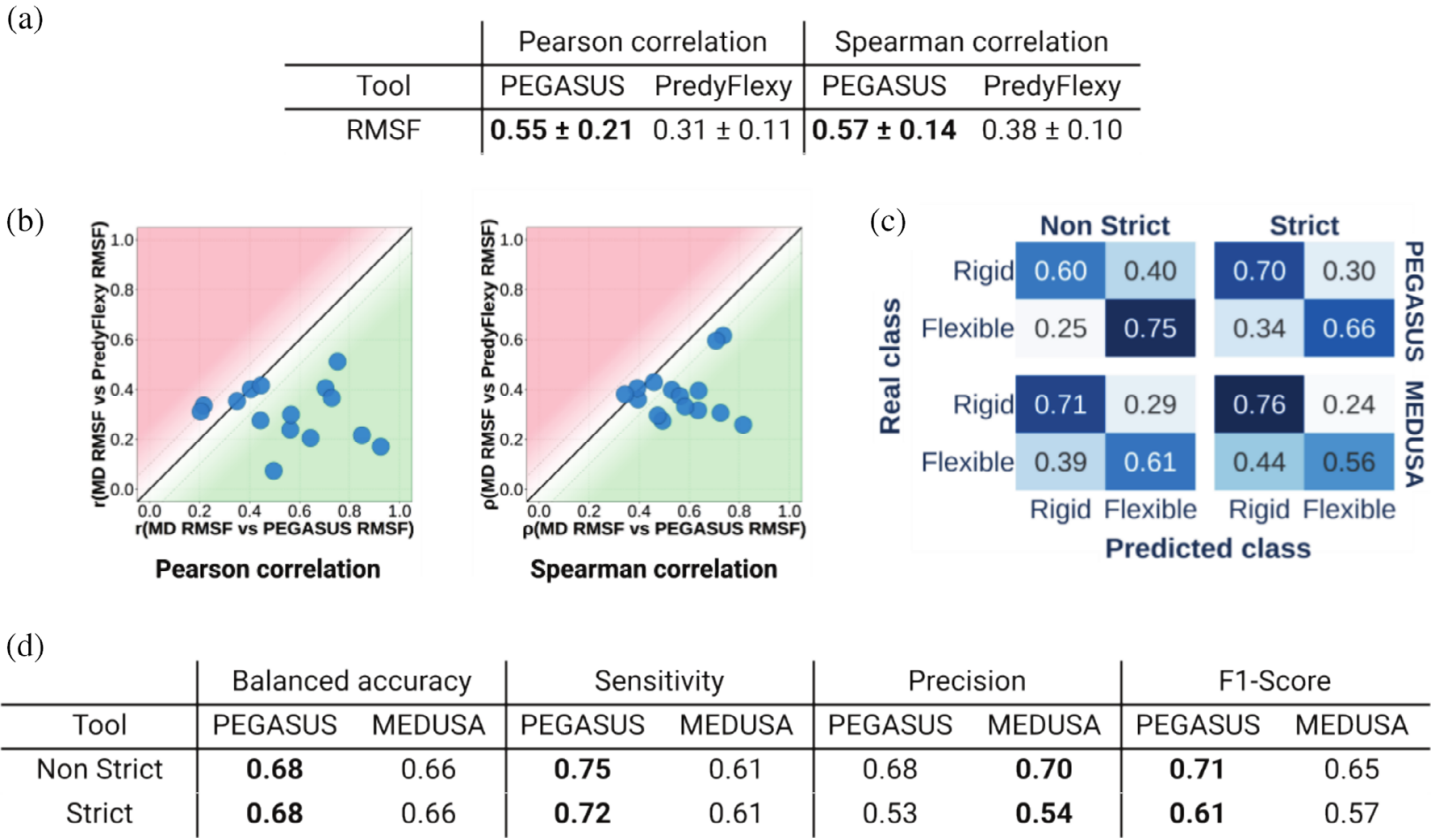
ProtEin lanGuAge models for prediction of SimUlated dynamicS (PEGASUS) performance comparison to state‐of‐the‐art methods. (a and b) Comparison of root mean square fluctuation (RMSF) prediction performance of PEGASUS and PredyFlexy. (a) Protein‐wise correlation between PEGASUS and PredyFlexy predictions against ground truth RMSF values, calculated for molecular dynamics (MD) simulations of the 15 free‐modeling targets from CASP15 dataset. (b) Comparison of the Pearson (left) and Spearman (right) correlations between RMSF measurements predicted with PEGASUS and MD RMSF (*X*‐axis), and Pearson correlations between PredyFlexy RMSF and MD RMSF (*Y*‐axis). Points in the green zone indicate that the PEGASUS predictions correlate better with MD RMSF rather than with PredyFlexy predictions. (c and d) Performance of PEGASUS for prediction of B‐factor class (0 stands for class “Rigid” and 1 for “Flexible”). (c) Confusion matrices of PEGASUS and MEDUSA performances for Strict and Non‐strict B‐factor class thresholds, on the CASP15 dataset. (d) Associated detailed comparison of the prediction metrics.

For RMSF prediction, PEGASUS demonstrates performance gain by 24% and 19% in terms of Pearson and Spearman correlation, respectively, as compared to PredyFlexy (Figure 1a). Moreover, PEGASUS predictions achieve correlation values above 0.8 for certain proteins, while protein‐wise correlation for PredyFlexy predictions hardly exceeds 0.5 (Figure 1b).

The PEGASUS protocol adaptation to the problem of protein flexibility prediction in two classes of B‐factor also provides an improvement for the majority of the model performance metrics as compared to the state‐of‐the‐art tool MEDUSA (Vander Meersche et al., 2021) (Figure 1c,d). Importantly, the train set used for MEDUSA development is more than seven times bigger than that of PEGASUS (9880 against 1369 proteins). The difference in performances achieved by the two methods comes from the particular efficiency of information‐rich pLM embeddings as compared to classically used evolutionary and physico‐chemical descriptors. Indeed, in MEDUSA, each residue is encoded by a 15‐residue sliding window (seven positions on each side), and each position contributes 100 features: (i) a 20‐dimensional one‐hot encoding of the amino acid identity, (ii) 58 physico‐chemical indices from the AAindex database (e.g., hydrophobicity, side‐chain volume, isoelectric point, etc.), and 21 evolutionary profile values (obtained using position‐specific scoring matrix for 20 amino acids and gap frequency) generated from HHblits search. pLM embeddings used in PEGASUS each provide between 768 and 2560 learned features per residue position that capture long‐range sequence patterns implicitly containing structural and functional information. This richer representation explains the better performance of PEGASUS over MEDUSA despite the smaller training set.

### 2.2 PEGASUS consistently and accurately predicts diverse MD‐derived flexibility metrics both for the ATLAS dataset and CASP15 free‐modeling targets

According to the results of 10‐fold cross‐validation on the ATLAS dataset, we observe strong Pearson correlations across all considered flexibility metrics, with low variability between folds (Table 1). We observe a particularly strong correlation for RMSF values as well as for Phi and Psi angle fluctuations (Table 1). For Mean LDDT, our model performance appears slightly lower, especially in terms of Spearman correlation. However, it still stays close to the MD‐derived values, with a very small Mean Absolute Error (MAE) of 0.07 out of 1. Moreover, the quality of predictions is very similar for different amino acid types and secondary structure types for all the flexibility metrics (Figures S10–S19).

**TABLE 1:** ProtEin lanGuAge models for prediction of SimUlated dynamicS performance on different metrics for 10‐fold cross‐validation performance on the ATLAS dataset and CASP15 free‐modeling (FM) target dataset. For the ATLAS dataset, we show the mean and standard deviation of the average values of Pearson and Spearman Correlation Coefficient (CC) as well as Mean Absolute Error (MAE) obtained in each cross‐validation fold. For the CASP15 dataset, we report the mean and standard deviation over 15 evaluated proteins. Several examples of the comparison of predicted and molecular dynamics‐derived flexibility profiles are given in Figures S24–S34.

	ATLAS			CASP15 FM		
	Pearson CC	Spearman CC	MAE	Pearson CC	Spearman CC	MAE
RMSF	0.75 ± 0.02	0.66 ± 0.02	0.82 ± 0.06 Å	0.69 ± 0.23	0.62 ± 0.14	1.57 ± 0.99 Å
Std. Phi	0.68 ± 0.01	0.73 ± 0.01	4.81 ± 0.18°	0.63 ± 0.15	0.63 ± 0.15	5.75 ± 1.00°
Std. Psi	0.66 ± 0.02	0.73 ± 0.01	5.68 ± 0.27°	0.63 ± 0.18	0.65 ± 0.13	6.54 ± 1.80°
Mean LDDT	0.63 ± 0.02	0.52 ± 0.02	0.07 ± 0.00	0.65 ± 0.21	0.56 ± 0.17	0.08 ± 0.04

Abbreviations: MAE, Mean Absolute Error; Mean LDDT, average Local Distance Difference Test; RMSF, root mean square fluctuation; Std. Phi, Phi angle standard deviation; Std. Psi, Psi angle standard deviation.

**FIGURE 2:**
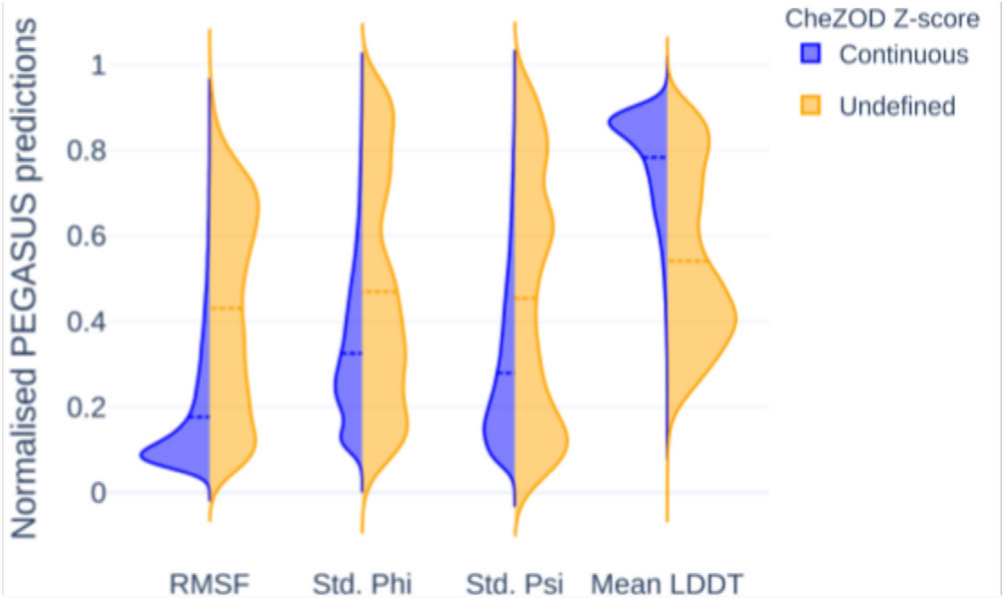
Distribution of ProtEin lanGuAge models for prediction of SimUlated dynamicS (PEGASUS) predictions for residues annotated and undefined in CheZOD database. Values are normalized between 0 and 1 for visualization. Mean LDDT, average Local Distance Difference Test; RMSF, root mean square fluctuation; Std. Phi, Phi angle standard deviation; Std. Psi, Psi angle standard deviation.

Our observations are confirmed by PEGASUS performance for free‐modeling targets of CASP15 dataset. Indeed, we observe a drop in correlation values by up to 10%, with RMSF remaining the best predicted flexibility descriptor (Table 1). CASP15 free‐modeling (FM) targets are particularly challenging for structure prediction (due to rare and uncommon folds in this category), which is consistent with the observed drop in the PEGASUS performance.

### 2.3 PEGASUS predictions can be used for evaluation of disorder

While PEGASUS was trained to predict flexibility of globular proteins, the flexibility metrics predicted for intrinsically disordered proteins of CheZOD dataset (chemical shift Z‐score for quantitative protein order and disorder assessment, Dass et al., 2020) correlate with the provided experimental evaluation of disorder degree (Figure 2, Table 2). Additionally, PEGASUS shows relevant results for the residues excluded from correlation calculations due to undefined NMR chemical shift *Z*‐scores (CheZOD scores) dataset. Indeed, these residues are mostly located at protein extremities or within low‐complexity regions, and likely correspond to highly disordered areas. Interestingly, although some of these residues exhibit higher predicted flexibility than annotated regions, distribution of PEGASUS predictions for residues with CheZOD *Z*‐scores classified as “Undefined” shows a pronounced tendency toward higher RMSF, Phi and Psi angle standard deviation (Std. Phi and Std. Psi) values and lower LDDT scores compared to regions with *Z*‐scores classified as “Continuous” (Figure 2). This finding suggests that PEGASUS not only successfully predicts disordered residues regardless of amino acid type (Figures S20–S23) but also provides additional insights for the regions where experimental measurements are not yet available and/or impossible to perform using available experimental settings. While dedicated disorder predictors benchmarked in CAID (Critical Assessment of Intrinsic protein Disorder, Necci et al., 2021) excel at identifying extended intrinsically disordered regions, PEGASUS provides a complementary, continuous‐valued perspective on residue mobility based on MD‐derived metrics, and can additionally be used to indicate potential disordered regions as a downstream application.

**TABLE 2:** Absolute values of correlation coefficients between ProtEin lanGuAge models for prediction of SimUlated dynamicS flexibility predictions and continuous disorder measurements from CheZOD dataset. Negative correlation is observed for CheZOD values versus root mean square fluctuation (RMSF), Phi and Psi angle standard deviation (Std. Phi, and Std. Psi). CheZOD score positively correlates with average Local Distance Difference Test (Mean LDDT) values.

	Pearson correlation	Spearman correlation
RMSF	0.61 ± 0.29	0.52 ± 0.25
Std. Phi	0.59 ± 0.24	0.54 ± 0.22
Std. Psi	0.62 ± 0.25	0.55 ± 0.22
Mean LDDT	0.65 ± 0.28	0.54 ± 0.25

### 2.4 Mean LDDT values predicted by PEGASUS correlate with AlphaFold 2 pLDDT but are not equivalent

**FIGURE 3:**
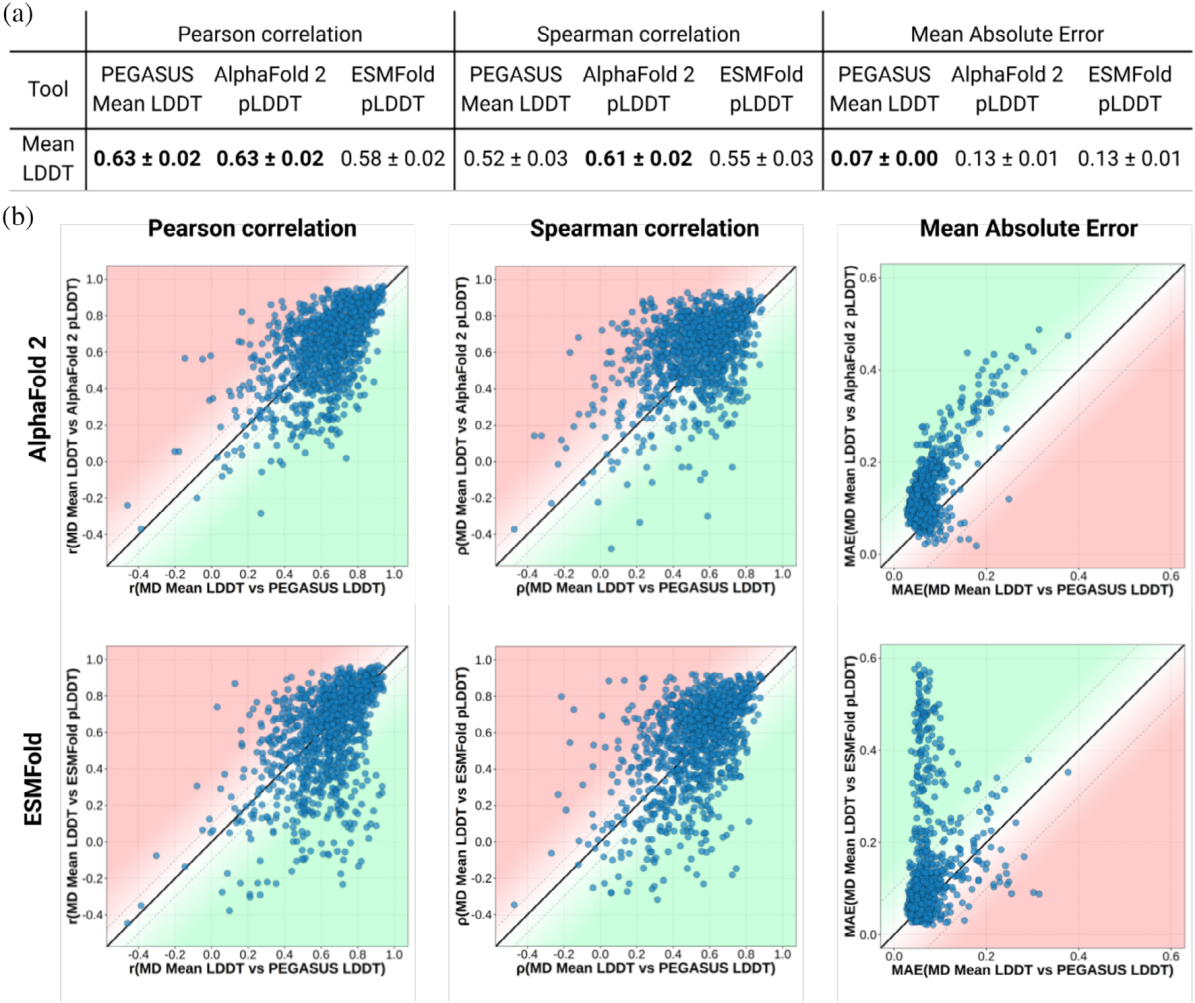
Evaluation of ProtEin lanGuAge models for prediction of SimUlated dynamicS (PEGASUS)'s average Local Distance Difference Test (Mean LDDT) prediction performance compared to AlphaFold 2's predicted Local Distance Difference Test (pLDDT), using 10‐fold cross‐validation on the ATLAS dataset. (a) Comparison of the performance of PEGASUS, AlphaFold 2 and ESMFold in representing Mean LDDT. (b) Top: Comparison of Pearson correlation, Spearman correlation, and Mean Absolute Error (MAE) between the Mean LDDT predicted by PEGASUS and the molecular dynamics (MD) ground truth (*X*‐axis) versus those between AlphaFold 2's pLDDT values and the MD ground truth (*Y*‐axis). Data points in the green zones indicate cases where PEGASUS's predictions are closer to the actual MD measurements than AlphaFold 2's pLDDT. Bottom: The same comparisons as above but using ESMFold's pLDDT instead of AlphaFold 2's pLDDT.

To assist users in evaluating the quality of structural predictions, the AlphaFold 2 team developed predicted local distance difference test (pLDDT) (Jumper et al., 2021). This metric corresponds to the expected LDDT (Mariani et al., 2013) between a predicted structure and the ground truth. Despite only being trained on static structures, pLDDT has shown impressive correlations with measurements of disorder and flexibility observed experimentally (Akdel et al., 2022; Binder et al., 2022; Ruff & Pappu, 2021). In our case, AlphaFold 2 pLDDT values also correlate well with Mean LDDT calculated from MD simulations with correlation coefficient values very close to those of PEGASUS (Figure 3a). Other PEGASUS metrics are also compared in Supporting Information S1, section “Correlation of PEGASUS metrics with AlphaFold 2 pLDDT”.

Nevertheless, Mean LDDT predicted by PEGASUS remains closer to the MD‐derived values in terms of mean average error. Moreover, a closer look at per‐protein predictions (Figure 3b, top) highlights a complementary nature of AF2 pLDDT and PEGASUS LDDT values. Indeed, performances between the tools vary significantly for a range of proteins, underscoring the complexity of the problem and suggesting the usage of a combination of both tools for consensus LDDT evaluation. Finally, ESMFold's pLDDT (Lin et al., 2023) clearly demonstrates a worse correlation with Mean LDDT values from MD simulations.

### 2.5 Case studies: PEGASUS Predictions can predict changes in flexibility upon point mutation

#### 2.5.1 p53 destabilizing mutation

**FIGURE 4:**
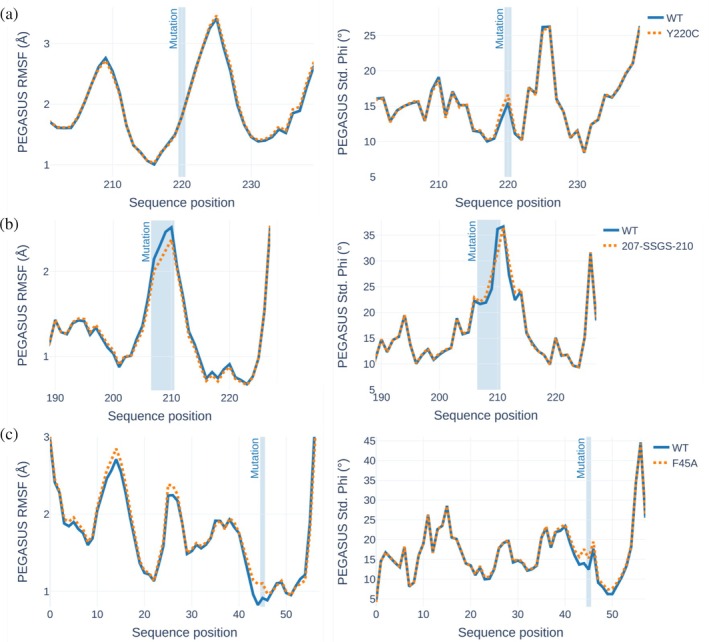
Evaluation of mutation impacts on the flexibility of three proteins (p53, GH11 xylanase XynCDBFV, and bovine pancreatic trypsin inhibitor) based on ProtEin lanGuAge models for prediction of SimUlated dynamicS (PEGASUS) predictions. Only root mean square fluctuation (RMSF) and Phi angle standard deviation (Std. Phi angles) are shown. Wild type (WT) and mutant forms are compared for each protein, with the mutation regions highlighted in light blue. (a) p53—comparison of WT and Y220C‐mutated forms. (b) GH11 xylanase XynCDBFV—comparison of WT and 207‐SSGS‐210‐mutated forms. (c) Bovine pancreatic trypsin inhibitor—comparison of WT and F45A‐mutated forms. Complete plots for other flexibility descriptors are provided in Figures S3–S5.

The p53 protein plays a critical role in regulating the cell cycle and apoptosis, and its mutations are commonly associated with various cancers (Hernández Borrero & El‐Deiry, 2021). One well‐studied mutation is Y220C, located in the DNA‐binding domain, which has been experimentally shown to disrupt the protein's thermostability (Basse et al., 2010). According to PEGASUS predictions, this mutation also leads to increased local flexibility of the mutation site in terms of Phi/Psi angle fluctuations without any large‐scale alteration of the RMSF and Mean LDDT flexibility profile (Figure 4a, Table S8), therefore suggesting a localized impact of the point mutation. Interestingly, another destabilizing mutation G245S has been reported to rigidify loop L3 (r234–r250) by MD simulations (Lepre et al., 2017). This was correctly captured by PEGASUS, which predicted a decrease in local flexibility (Std. Phi) between r240 and r245 (Figure S6).

#### 2.5.2 Thermostable mutant of GH11 xylanase (XynCDBFV)

Xylanase enzymes are widely used in industrial applications such as paper processing and animal feed production, creating a demand for more thermostable variants for optimized biotechnological treatments (Kumar et al., 2017). One such variant is the 207‐SSGS‐210 mutant of GH11 xylanase XynCDBFV, which is known to be a more thermostable form of the enzyme (Han et al., 2017). PEGASUS successfully identifies the rigidification of the corresponding protein region noticeable both for Phi and Psi angle fluctuations as well as for RMSF (Figures 4b and S4). In Figure S7, we explore another example of the RMSF prediction for RMSF of the GH11 xylanase with two destabilizing mutations A210S and G208S. For the A210S mutation in particular, we observe a marked increase in RMSF at the site of mutation (Cheng, Chen, et al., 2014).

#### 2.5.3 Destabilizing mutation of bovine pancreatic trypsin inhibitor (BPTI)

BPTI is a small, well‐studied protein that plays a critical role in regulating the activity of enzymes like trypsin protease. Several mutations, such as F45A, have been shown to destabilize the protein (Kim et al., 1993). PEGASUS predictions indicate destabilization of the mutated protein region according to all metrics (Figure 4c), with a large part of the C‐terminus region impacted by mutation according to the Mean LDDT profile (Figure S5). Moreover, two additional peaks at positions 14 and 26 in the RMSF profile suggest a long‐range effect of destabilizing mutations. Moreover, a similar increase in RMSF values is observed for the mutation at the close position N43G, which is in accordance with its reported destabilizing effect (Danishefsky et al., 1993) (Figure S8).

Even though predicted changes in flexibility profiles in response to mutations appear rather small, we find that in many cases the predictions of PEGASUS are in line with previous experimental observations, suggesting its potential for exploration of the expected effect of point mutations on protein stability.

### 2.6 PEGASUS web server and standalone utility provide versatile tools for protein flexibility prediction and large‐scale comparison of the results

Optimized protocol for pLM embedding generation and deep learning (DL)‐based predictions (see Section 4 for the details) allows us to obtain unprecedentedly fast prediction results even for proteins of substantial length (up to 100 sequences of 1 k residues simultaneously). Furthermore, the user has a possibility to submit batches of hundreds of protein sequences and receive predictions within several minutes (see Figure S9 for the computation time evaluation). The user can also provide a protein sequence alignment as input to the web server and visualize the predicted flexibility profiles in accordance with the alignment. Such an additional option further expands biological applications of PEGASUS for such important tasks as large‐scale mutagenesis analysis or comparison of protein flexibility for families of homologous proteins.

## 3 DISCUSSION

Prediction of protein dynamic properties remains one of the major challenges in structural bioinformatics following a breakthrough in computational 3D structure prediction. PEGASUS developed in the current study provides a fast and efficient tool for the prediction of protein flexibility descriptors directly from amino acid sequences. As we demonstrate above, PEGASUS clearly outperforms state‐of‐the‐art tools as well as provides a more detailed view of the expected protein dynamics using several descriptors. Moreover, PEGASUS predictions are well correlated with experimentally observed structural variability of intrinsically disordered proteins and complement information on local protein deformability as can be assessed by pLDDT AlphaFold 2 values. Notably, PEGASUS operates significantly faster thanks to its lightweight architecture, making it a possible alternative to AlphaFold 2 pLDDT for flexibility estimation, especially for large‐scale studies where computational efficiency is crucial.

Development of PEGASUS was possible thanks to the recent release of powerful pLMs. Information‐rich pLM embeddings allow efficient training of predictive models on datasets of limited size, which is particularly important for our task. Moreover, our study highlights the advantage of the combination of predictors trained on different pLM models, likely due to the inherent diversity among pLMs. Nevertheless, while PEGASUS infers flexibility indirectly from sequence embeddings, which may encode structural information implicitly, it should not be used as a substitute for an explicit structural model.

Further improvement of the developed method would imply augmentation and diversification of the source data. Since all our training data are derived from 100 ns MD simulations, PEGASUS is optimized for local and moderate global motions that occur on this timescale; it may not fully capture rare and large conformational changes. Future extensions of the ATLAS dataset to longer simulations (~1 μs) or enhanced‐sampling trajectories would broaden PEGASUS applicability to such processes. Furthermore, the quality of predictions is impacted by protein length (Figure S1): small proteins tend to show higher error rates, possibly due to their general higher deformability, while large proteins are generally less prone to significant prediction errors, but may not achieve the highest correlation coefficients (Figure S2), suggesting that more data would allow prediction improvement for extreme cases. Based on the ATLAS dataset, PEGASUS predictions are only reliable for globular protein dynamics in solution with no partner contacts. Access to protein multimer simulations, data on membrane protein dynamics, as well as protein dynamics in the presence of ligands is crucial for further expansion of PEGASUS applications. Finally, all the descriptors were derived from 100 ns MD simulations, which may not be long enough to detect large‐scale conformational changes. According to the *Dynamic PDB* study (Liu et al., 2024), while more reliable on average, no significant changes in RMSF values are observed in 1 μs simulations as compared to 100 ns ones. However, the incorporation of larger proteins and protein ensembles would require further extension of the simulation time. These issues are partially addressed by an upcoming update of the ATLAS dataset (Vander Meersche et al., 2024) and mdCATH database (Mirarchi et al., 2024) but a qualitative leap forward in predictive model performance would require the incorporation of even more diverse datasets hopefully made available thanks to the continuous effort of the community on MD data production and sharing (Amaro et al., 2024; Beltrán et al., 2024; Mirarchi et al., 2024; Siebenmorgen et al., 2024; Tiemann et al., 2024).

## 4 MATERIALS AND METHODS

### 4.1 Training dataset

The flexibility metrics used for predictions were derived from the ATLAS dataset (Vander Meersche et al., 2024), which consists of 1390 standardized all‐atom MDs simulations, representative of the structural space of globular proteins. Each protein was simulated in three replicates of 100 ns, in all‐atom simulations with explicit solvent under a unique protocol (Vander Meersche et al., 2024). The MD conformations were aligned to the first frame using a least‐squares fit on the backbone atoms with GROMACS. To enhance computational efficiency, proteins with more than 800 amino acids were excluded, resulting in a final dataset of 1369 proteins, encompassing a total of 306,819 amino acids.

### 4.2 Flexibility measurements

To capture the complexity of protein motion, four different metrics are predicted:

#### 4.2.1 Root mean square fluctuation (in Å)

RMSF measures the standard deviation of an atom's position throughout the trajectory, offering a view of overall flexibility. It is calculated on α‐carbons using GROMACS (Abraham et al., 2015).

#### 4.2.2 Average Local Distance Difference Test

Mean LDDT represents the average LDDT (Mariani et al., 2013) values across each frame of the trajectory, with the first frame as the reference. This metric, calculated on α‐carbons using OpenStructure's LDDT executable (Biasini et al., 2013) with default parameters, provides a more global assessment of dynamics by considering variations in neighboring internal contacts and is not influenced by the alignment step required for RMSF calculations.

#### 4.2.3 Phi and Psi angle standard deviation (Std. Phi and Std. Psi) (in°)

These metrics represent the standard deviation of the backbone torsion angles (Phi and Psi) over MD frames, providing insight into the local deformability of the protein backbone. The angles are calculated using MDTraj (McGibbon et al., 2015). Periodicity of Phi/Psi angles was taken into account while calculating mean and standard deviation values by using the functions circmean and circstd from the scipy Python package.

Flexibility measurements from the ATLAS dataset were averaged across three MD replicates, using 10,000 MD frames for each 100 ns replicate. Mean LDDT was calculated using only 1000 frames due to the large size of the dataset and associated computational limitations.

### 4.3 Protein sequence encoding using protein language models

Protein sequences of the ATLAS dataset are encoded using information‐rich protein embeddings generated by pLMs. These embeddings are numerical representations of protein sequences, created by deep learning models trained on millions of sequences to predict hidden parts of the sequence based on the surrounding context. Embeddings are therefore highly effective for tasks like predicting protein structure (Jumper et al., 2021; Lin et al., 2023), function (Yuan et al., 2023), or disorder (Ilzhöfer et al., 2022), generally outperforming traditional multiple sequence alignment methods while being generated much more quickly. It is important to note that pLMs, used in methods such as ESMFold, rely solely on single‐sequence embeddings and underperform against methods that also exploit evolutionary information, such as AlphaFold 2. Nevertheless, pLM‐only embeddings can outperform classical features derived from multiple sequence alignment (MSA) when used in equivalent network architectures (Erckert & Rost, 2024; Littmann et al., 2021). Indeed, hybrid approaches such as AlphaFold 2 Evoformer, which apply Transformer‐based feature learning directly to MSAs, achieve the strongest results by combining both sources of information.

**FIGURE 5:**
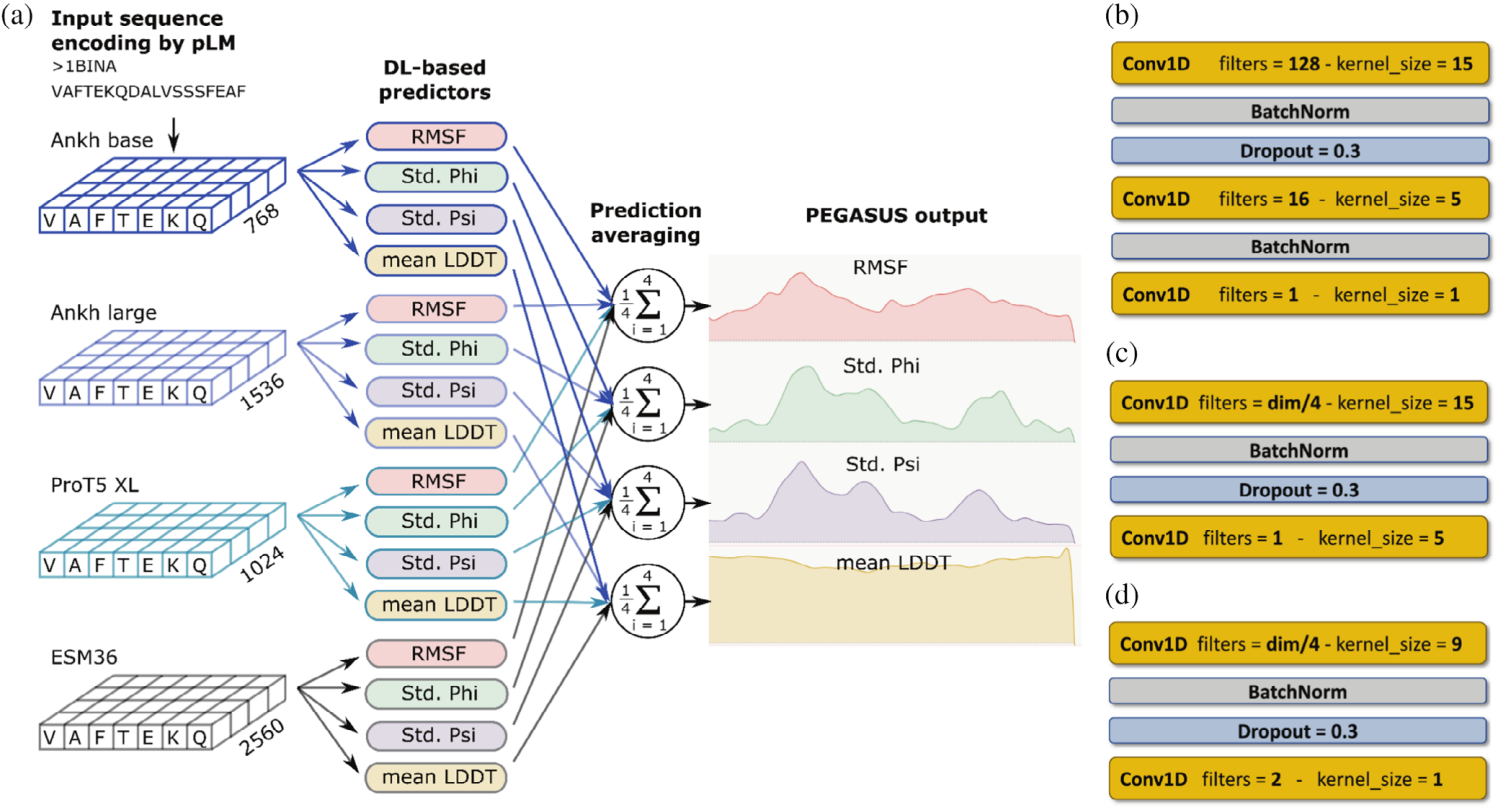
ProtEin lanGuAge models for prediction of SimUlated dynamicS (PEGASUS) prediction pipeline. (a) Main steps of the PEGASUS prediction protocol. (b) Architecture of DL‐based models for root mean square fluctuation (RMSF) and average Local Distance Difference Test (Mean LDDT) prediction. (b) Architecture of DL‐based models for Phi angle standard deviation (Std. Phi) and Psi angle standard deviation (Std. Psi) prediction. (c) Architecture of DL‐based models for B‐factor class prediction (used to compare with MEDUSA). Further details on neural network architectures are provided in Tables S1–S7. In (b and c) “dim” stands for the dimension of the corresponding protein Language Models (pLM) embedding (specified in panel a).

We used the following embeddings to encode protein sequence in PEGASUS: the Base and Large Ankh models (Elnaggar et al., 2023), the ESM36 model (Rives et al., 2021), and the ProtT5 XL UniRef50 model (Elnaggar et al., 2022). These models encode each sequence position with 768, 1536, 2560, and 1024 features, respectively. Each pLM embedding was used as an input to train a corresponding predictor. For each flexibility descriptor, the final model prediction corresponded to an average among four output values predicted by individual pLM‐based models. The resulting ensemble model outperforms models based on each of the pLM embeddings taken individually, as well as models based on a combination of fewer predictors (for the details, see Supporting Information S1, section “Model selection,” Tables S1–S3). Moreover, the addition of ESM48 embeddings provides only marginal improvement of the model performance while drastically increasing the number of trainable parameters (Table S4) and therefore was excluded from the final pipeline. Our results confirm a previous observation that the combination of pLM‐based predictors shows a greater performance in downstream tasks (Chua et al., 2024; Pokharel et al., 2023). The full PEGASUS pipeline is given in Figure 5.

### 4.4 Architecture of pLM‐based predictors

Given the specific requirements of each flexibility metric, four distinct predictive pipelines were developed and optimized independently. This process led to the selection of two shallow convolutional neural network (CNN) architectures: a three‐layer CNN for predicting RMSF and Mean LDDT, and a two‐layer CNN for predicting Std. Phi and Std. Psi angles (Figure 1). In total, 16 neural network models were trained. The combination of convolution filters with pLM‐based sequence representation enables the network to capture long‐range interactions. For the sake of computational efficiency, each model takes a complete sequence embedding as an input. The architecture choices were guided by previous research on embedding‐based prediction tasks, showing that shallow CNNs are often very effective for extracting valuable information from already information‐rich embeddings (Bernhofer & Rost, 2022; Ilzhöfer et al., 2022).

Model weights were optimized using a mean squared error (MSE) loss function and Adam optimizer. We also identify the optimal hyperparameters, such as learning rate, batch size, number of epochs, and CNN layer configuration by testing various combinations through a grid‐search approach for each of the 16 networks in 10‐fold cross‐validation. All models were implemented and trained in Python (v3.10.8) using PyTorch (v1.13.0) (Paszke et al., n.d.).

### 4.5 Models performance evaluation

We evaluated prediction performance using a 10‐fold cross‐validation process to ensure an unbiased assessment. Given the varying prevalence of Evolutionary Classification of protein Domains (ECOD) domains (Cheng, Schaeffer, et al., 2014) in the ATLAS dataset (Vander Meersche et al., 2024), we additionally clustered the domains by homology level (H‐class) into 10 subsets, each containing a balanced number of H‐classes and total number of domains. These subsets were then used to create the cross‐validation folds, with each subset serving once for testing and validation, while the remaining eight subsets are used for training. To eliminate redundancy, multi‐domain proteins with ECOD domains overlapping the training set in any fold were excluded from the validation and test sets. This approach resulted in 1121 test proteins, with each fold containing between 95 and 128 proteins.

The similarity between predicted and true values was assessed using the Pearson and Spearman correlation coefficient, as well as MAE. Pearson correlation was used for descriptive assessment of linear agreement. We complement all analyses with Spearman correlation to ensure robustness to non‐normality and bounded data. Final model weights for each metric were chosen by comparing performance across the cross‐validation and selecting the fold with the best consistent correlation scores for all metrics.

### 4.6 Comparison with existing flexibility prediction methods

#### 4.6.1 Comparison with PredyFlexy

PEGASUS RMSF prediction performance was compared to that of PredyFlexy (de Brevern et al., 2012). To ensure a rigorous comparison, both methods were tested on an independent dataset curated from the CASP15 (Alexander et al., 2023) free‐modeling proteins, by manually selecting X‐ray crystallography structures with fewer than 40 consecutive missing residues and a resolution below 2.5 Å. This process yielded 15 proteins, none of which shared homology with the training data used by either PredyFlexy or PEGASUS. Both tools were used to predict protein flexibility through their respective web servers, and the predictions were compared to ground truth RMSF measurements. These RMSF values were obtained by performing MDs simulations using the same protocol as the one used for the generation of the ATLAS database (Vander Meersche et al., 2024). Missing residues were reconstructed using AlphaFold 2 (Jumper et al., 2021).

#### 4.6.2 Comparison with MEDUSA

To compare PEGASUS performance to that of the state‐of‐the‐art protein flexibility prediction tool MEDUSA (Vander Meersche et al., 2021), we have adapted the PEGASUS pipeline for prediction of protein flexibility in two classes of B‐factor values. We trained two models using strict and non‐strict thresholds of *Z*‐score normalized B‐factor values (Schlessinger et al., 2006). PEGASUS final prediction was provided by the average of four class probabilities predicted by different pLM‐based models.

Both strict and non‐strict networks were trained similarly to PEGASUS original models, but using cross‐entropy loss and class weighting to give more emphasis to the underrepresented flexible class. Performance was assessed using B‐factor values from our CASP15 (Alexander et al., 2023) free‐modeling dataset, using balanced accuracy, precision, sensitivity, and F1‐score measurements.

It is important to mention that while room‐temperature B‐factors better capture physiological dynamics, the predominance, higher precision, and standardized normalization of cryo‐temperature B‐factors in structural databases make them a meaningful and reproducible target for model training and benchmarking. Indeed, over 85% of high‐resolution crystal structures in the PDB are collected under cryogenic conditions. By restricting our analysis to these datasets, we both maximize sample size and minimize variability arising from differing experimental protocols. Moreover, as shown in (Carugo, 2022), the estimated error on individual B‐factors is substantially lower at cryogenic temperatures (~6 Å^2^) than at ambient temperature (~9 Å^2^). Although these values still include contributions from crystal defects and data‐quality issues, cryo‐collected B‐factors are measurably more precise, providing a more reproducible proxy for local disorder across different structures. Finally, to ensure fair comparisons, we applied per‐structure *Z*‐normalization of B‐factors to remove global scale differences (as recommended in Carugo, 2022), so that our machine‐learning predictors are trained and evaluated on relative fluctuations within each crystal.

### 4.7 Comparison with experimental disorder measurement

To assess the relevance of PEGASUS predictions to protein disorder, we used continuous disorder measurements from the CheZOD dataset (Dass et al., 2020). This dataset comprises 1325 proteins with an overall balanced content of disordered residues, derived from NMR. Disorder degree is evaluated from the difference between experimentally determined NMR secondary chemical shifts and computed random coil reference values (Nielsen & Mulder, 2020). More precisely, it is derived from the root mean squared deviation (RMSD) of all chemical shift residuals within a residue triplet and linear combinations of its fractional powers (Nielsen & Mulder, 2016). Residues with scores below 3 are considered to be disordered, partially formed/fractionally occupied structures have scores between 3 and 8, and fully formed structures correspond to scores above 8 (Nielsen & Mulder, 2016).

In our study, we removed sequences containing unknown amino acids (denoted as “X”), resulting in a dataset of 1316 proteins used for PEGASUS prediction.

### 4.8 Comparison with AlphaFold 2 and ESMFold pLDDT values

The pLDDT values from AlphaFold 2 for the ATLAS dataset's proteins were extracted from 3D structure predictions using the local version of AlphaFold 2 ColabFold v1.5.1 (Mirdita et al., 2022) with default settings, that is, MMseqs2 (Steinegger & Söding, 2017) for homology searches (UniRef30 + env), and a template search on the PDB70 via online Application Programming Interface (API). The prediction process includes three recycling steps, followed by GPU relaxation using OpenMM/Amber to generate five models, selecting the top‐ranked model based on the average pLDDT score.

The ESMFold pLDDT values were obtained from 3D structure predictions using the local version of ESMFold (Lin et al., 2023) with four recycling steps, without requiring any sequence alignments or template search, unlike AlphaFold 2.

### 4.9 Web server implementation: Fast pLM inference using API

To enable rapid flexibility predictions, we designed the PEGASUS web server with a client–server architecture that uses an asynchronous API for efficient pLM inference. This setup separates the computationally intensive embedding generation from the main application, enhancing performance and resource utilization.

The server comprises two main components: a front‐end application and a pLM inference API. The front‐end, implemented in Python, manages user inputs, prediction workflows, and result visualization. The back‐end API, built with FastAPI, generates sequence embeddings using pre‐trained pLMs.

Upon user submission of sequences, the front‐end asynchronously dispatches requests to the pLM inference API via the *aiohttp* library. The API supports the following pLMs: ProtT5‐XL‐UniRef50 (Liu et al., 2024), ESM2 (Hernández Borrero & El‐Deiry, 2021), and Ankh models (Ruff & Pappu, 2021). Asynchronous processing allows parallel handling of multiple sequences and models, significantly reducing computation time and optimizing resource usage, especially when multiple jobs are running in parallel.

Thread‐safe access to models in the API is ensured using *asyncio* locks, maintaining model integrity during concurrent inference. Models are loaded into memory once and kept ready to efficiently serve requests. After generating embeddings, the API returns them to the front end, which then uses PEGASUS pre‐trained CNNs for predictions of each flexibility metric. Such architecture offers several benefits. Offloading embedding generation reduces the front end's memory footprint and keeps it responsive. The use of asynchronous requests and parallel processing optimizes resource utilization, enabling the server to handle high‐throughput demands. The API is deployed on GPUs, which further accelerates computations.

Benchmark tests indicated that this implementation reduces average prediction time by approximately 90% compared to a synchronous cpu‐only approach (data not shown). Users can obtain flexibility predictions for up to 100 sequences of maximum 1000 residues simultaneously, facilitating large‐scale analyses.

The standalone version of PEGASUS is available at https://github.com/DSIMB/PEGASUS. Optionally, users can generate HTML result pages for each protein as well as the overview page in order to get the same interactive experience as on the web server.

## AUTHOR CONTRIBUTIONS

**Yann Vander Meersche:** Data curation; software; investigation; writing – original draft; validation; formal analysis; writing – review and editing. **Gabriel Duval:** Software; methodology; investigation; conceptualization; validation; data curation; formal analysis. **Gabriel Cretin:** Software; methodology; writing – review and editing; investigation; validation; data curation; resources. **Aria Gheeraert:** Investigation; writing – review and editing; validation; software. **Jean‐Christophe Gelly:** Conceptualization; investigation; writing – review and editing; visualization; validation; methodology; formal analysis; supervision. **Tatiana Galochkina:** Conceptualization; investigation; funding acquisition; writing – original draft; methodology; validation; writing – review and editing; formal analysis; project administration; supervision; visualization.

## Supporting information


**Data S1.** Supporting Information.

## Data Availability

The data that support the findings of this study are openly available in Atlas of proTein moLecular dynAmicS at https://www.dsimb.inserm.fr/ATLAS.
