# Supplementary material for: PEGASUS: Prediction of MD‐derived protein flexibility from sequence
Source: Protein Sci. 2025 Jul 16;34(8):e70221. doi: 10.1002/pro.70221 (PMC12267886; doi:10.1002/pro.70221)
Supplement: Supplementary file 1 — Data S1. Supporting Information. [file PRO-34-e70221-s001.pdf]

PEGASUS: Prediction of MD-derived protein flexibility from sequence

SUPPLEMENTARY DATA

Yann Vander Meersche[#,1], Gabriel Duval[#,1], Gabriel Cretin[#,1], Aria Gheeraert[1], Jean-Christophe Gelly[*,1], Tatiana Galochkina[*,1]

[#] These authors contributed equally.

* Corresponding authors: jean-christophe.gelly@u-paris.fr, tatiana.galochkina@u-paris.fr

[1] Université Paris Cité and Université des Antilles and Université de la Réunion, INSERM, BIGR, DSIMB, F-75015 Paris, France

Model selection 1
PEGASUS predictions and protein size 5
Final model architecture and number of parameters 7
Correlation of PEGASUS metrics with AlphaFold 2 pLDDT 7
PEGASUS point mutation prediction 8
Web server implementation 11
Performance evaluation for different types of amino acids: ATLAS dataset 13
Comparison with degree of protein disorder for different amino acid types: CheZOD dataset 18
Top-Ranked Proteins by Correlation and Error Metrics 20

Model selection

Protein sequence encoding using protein language models combined to shallow convolutional neural networks has demonstrated its efficiency for a range of different prediction tasks. Moreover, as it was demonstrated in previous works such as ProtGOAT [1], ensemble models based on combination of the predicted output by models trained on different pLM embeddings can further increase prediction performance. Therefore, to choose an optimal model for protein flexibility prediction we tested combinations ensembles of predictive models trained on the commonly used pLM embeddings:

- Ankh (base and large model) [2]
- ESM (36 and 48) [3]
- ProtT5 XL UniRef50 [4]

First, we have performed our model choice for RMSF value prediction using a single independent test set filtered by structural similarity. The significance of the observed difference in the ensemble model performance versus that obtained by combinations of lower number of models was estimated using p-values calculated using a Bonferroni-corrected T-test.

Table S1: Comparison of the ensemble model performance for five different embeddings (first line) to the performance of five models using individual embeddings. For each line we show in green pLM-based models used for prediction. We highlight in bold best values of Spearman and Pearson coefficients on the test dataset as well as p-values below 1E-01.

pLM embedding used by a predictive model					Correlation coefficient		Corrected p-values	
AnkhL	AnkhB	ESM36	ESM48	ProtT5	Spearman	Pearson	Spearman	Pearson
+	+	+	+	+	**0.68**	**0.76**	-	
-	-	-	-	+	0.49	0.65	**1.79E-132**	**1.31E-42**
-	-	-	+	-	0.59	0.70	**2.76E-34**	**2.60E-16**
-	-	+	-	-	0.58	0.69	**2.98E-42**	**3.05E-19**
-	+	-	-	-	0.57	0.68	**6.64E-53**	**3.98E-23**
+	-	-	-	-	0.58	0.69	**2.99E-47**	**4.40E-21**

Table S2: Comparison of the ensemble model performance for five different embeddings (first line) to the performance of ensemble models including using two different pLM embeddings. For each line we show in green pLM-based models used for prediction. We highlight in bold best values of Spearman and Pearson coefficients on the test dataset as well as p-values below 1E-01.

pLM embedding used by a predictive model					Correlation coefficient		Corrected p-values	
AnkhL	AnkhB	ESM36	ESM48	ProtT5	Spearman	Pearson	Spearman	Pearson
+	+	+	+	+	**0.68**	**0.76**	-	
-	-	-	+	+	0.59	0.72	**1.53E-38**	**1.58E-07**
-	-	+	-	+	0.59	0.72	**1.60E-35**	**4.34E-08**
-	+	-	-	+	0.59	0.72	**3.39E-35**	**2.30E-09**
+	-	-	-	+	0.60	0.72	**1.07E-31**	**2.19E-08**
-	-	+	+	-	0.63	0.73	**1.86E-10**	**7.06E-05**
-	+	-	+	-	0.635	0.73	**5.45E-09**	**7.48E-04**
+	-	-	+	-	0.635	0.74	**2.46E-09**	**2.61E-03**
-	+	+	-	-	0.63	0.73	**1.85E-11**	**4.15E-05**
+	-	+	-	-	0.63	0.734	**8.75E-10**	**7.33E-04**
+	+	-	-	-	0.63	0.73	**2.08E-12**	**7.85E-06**

Table S3: Comparison of the ensemble model performance for five different embeddings (first line) to the performance of ensemble models including using three different pLM embeddings. For each line we show in green pLM-based models used for prediction. We highlight in bold best values of Spearman and Pearson coefficients on the test dataset as well as p-values below 1E-01.

pLM embedding used by a predictive model					Correlation coefficient		Corrected p-values	
AnkhL	AnkhB	ESM36	ESM48	ProtT5	Spearman	Pearson	Spearman	Pearson
+	+	+	+	+	**0.68**	**0.76**	-	
-	-	+	+	+	0.63	0.74	**1.12E-10**	7.25E-02
-	+	-	+	+	0.635	0.74	**2.16E-09**	1.05E-01
+	-	-	+	+	0.64	0.75	**2.14E-08**	2.09E-01
-	+	+	-	+	0.64	0.74	**4.70E-09**	**3.66E-02**
+	-	+	-	+	0.64	0.745	**1.45E-07**	1.29E-01
+	+	-	-	+	0.64	0.74	**9.08E-09**	**1.69E-02**
-	+	+	+	-	0.66	0.75	**5.78E-02**	3.96E-01
+	-	+	+	-	0.66	0.75	1.04E-01	1.00E+00
+	+	-	+	-	0.66	0.75	1.66E-01	7.89E-01
+	+	+	-	-	0.66	0.75	8.28E-02	3.54E-01

Table S4: Comparison of the ensemble model performance for five different embeddings (first line) to the performance of ensemble models including using four different pLM embeddings. For each line we show in green pLM-based models used for prediction. We highlight in bold best values of Spearman and Pearson coefficients on the test dataset as well as p-values below 1E-01.

pLM embedding used by a predictive model					Correlation coefficient		Corrected p-values	
AnkhL	AnkhB	ESM36	ESM48	ProtT5	Spearman	Pearson	Spearman	Pearson
+	+	+	+	+	**0.68**	**0.76**	-	
+	+	+	+	-	0.66	0.75	2.06E-01	4.94E-01
+	+	+	-	+	0.66	0.75	3.95E-01	9.05E-01
+	+	-	+	+	0.66	0.74	1.19E-01	1.08E-01
+	-	+	+	+	0.65	0.75	7.40E-02	3.89E-01
-	+	+	+	+	0.64	0.75	**4.72E-0.2**	2.58E-01

Table S5: Performance for the five embedding model against a variation with an added one-hot encoding (OH) and OH alone. For each line we show in green pLM-based models used for prediction. We highlight in bold best values of Spearman and Pearson coefficients on the test dataset as well as p-values below 1E-01.

pLM embedding used by a predictive model						Correlation coefficient		Corrected p-values	
AnkhL	AnkhB	ESM36	ESM48	ProtT5	OH	Spearman	Pearson	Spearman	Pearson
+	+	+	+	+	-	**0.68**	**0.76**	-	
+	+	+	+	+	+	0.67	0.76	1.00E+00	1.00E+00
-	-	-	-	-	+	0.44	0.60	**5.43E-153**	**2.77E-51**

According to our results, ensemble models based on five predictors using different pLM embeddings outperforms any pLM-based model using individual embedding and also outperforms any ensemble of two pLM-based models with statistically significant difference in both Spearman and Pearson correlation coefficients. The performance of the ensemble model based on five pLM predictors remains higher than that of any combination of three pLM-based predictors even though the difference is not always significant. Finally, we made a choice to exclude the pLM-based predictor using ESM48 embedding from the final model due to its important size significantly increasing calculation time without prominent improvement of the model performance.

In order to make sure that our conclusions can be extrapolated to other target variables than RMSF, we performed a performance comparison for pLM-based models using one embedding versus our final model (Fig. S1) using a complete ten-fold cross-validation on ATLAS dataset. PEGASUS outperforms the models based on individual pLM embeddings for all the considered flexibility descriptors.

Table S6: Comparison of PEGASUS performances against single pLM predictions. Results display 10-fold cross-validation PEGASUS predictions on the ATLAS dataset.

**RMSF (Å)**	Pearson correlation	Spearman correlation	Mean absolute error
Ankh Large	0.69 ± 0.02	0.58 ± 0.04	0.92 ± 0.08
Ankh Base	0.69 ± 0.02	0.57 ± 0.03	0.91 ± 0.08
ESM T36	0.69 ± 0.02	0.58 ± 0.02	0.89 ± 0.07
ProtT5	0.65 ± 0.03	0.49 ± 0.04	0.93 ± 0.06
PEGASUS	0.75 ± 0.02	0.66 ± 0.02	0.82 ± 0.06

**Mean LDDT**	Pearson correlation	Spearman correlation	Mean absolute error
Ankh Large	0.60 ± 0.02	0.50 ± 0.02	0.08 ± 0.01
Ankh Base	0.54 ± 0.08	0.43 ± 0.08	0.08 ± 0.01
ESM T36	0.57 ± 0.03	0.46 ± 0.03	0.07 ± 0.00
ProtT5	0.54 ± 0.02	0.42 ± 0.03	0.08 ± 0.00
PEGASUS	0.63 ± 0.02	0.52 ± 0.02	0.07 ± 0.00

**Std. Phi (°)**	Pearson correlation	Spearman correlation	Mean absolute error
Ankh Large	0.64 ± 0.02	0.68 ± 0.02	5.34 ± 0.19
Ankh Base	0.63 ± 0.01	0.67 ± 0.01	5.32 ± 0.16
ESM T36	0.64 ± 0.01	0.68 ± 0.01	5.61 ± 0.40
ProtT5	0.64 ± 0.01	0.67 ± 0.01	5.35 ± 0.15
PEGASUS	0.68 ± 0.01	0.73 ± 0.01	4.81 ± 0.18

**Std. Psi (°)**	Pearson correlation	Spearman correlation	Mean absolute error
Ankh Large	0.63 ± 0.02	0.66 ± 0.03	6.41 ± 0.32
Ankh Base	0.62 ± 0.01	0.67 ± 0.01	6.28 ± 0.25
ESM T36	0.62 ± 0.01	0.66 ± 0.02	6.53 ± 0.32
ProtT5	0.62 ± 0.02	0.66 ± 0.01	6.40 ± 0.22
PEGASUS	0.66 ± 0.02	0.73 ± 0.01	5.68 ± 0.27

PEGASUS predictions and protein size

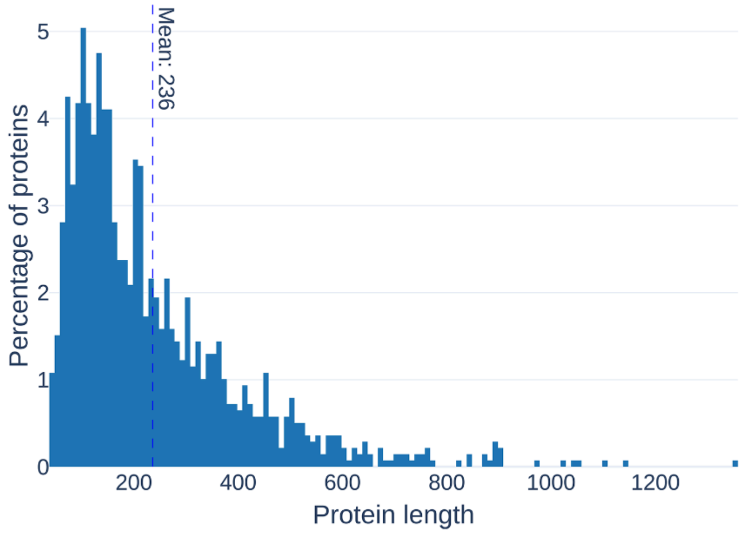

Figure S1: Distribution of ATLAS dataset's protein length (between 38 and 2128 residues).

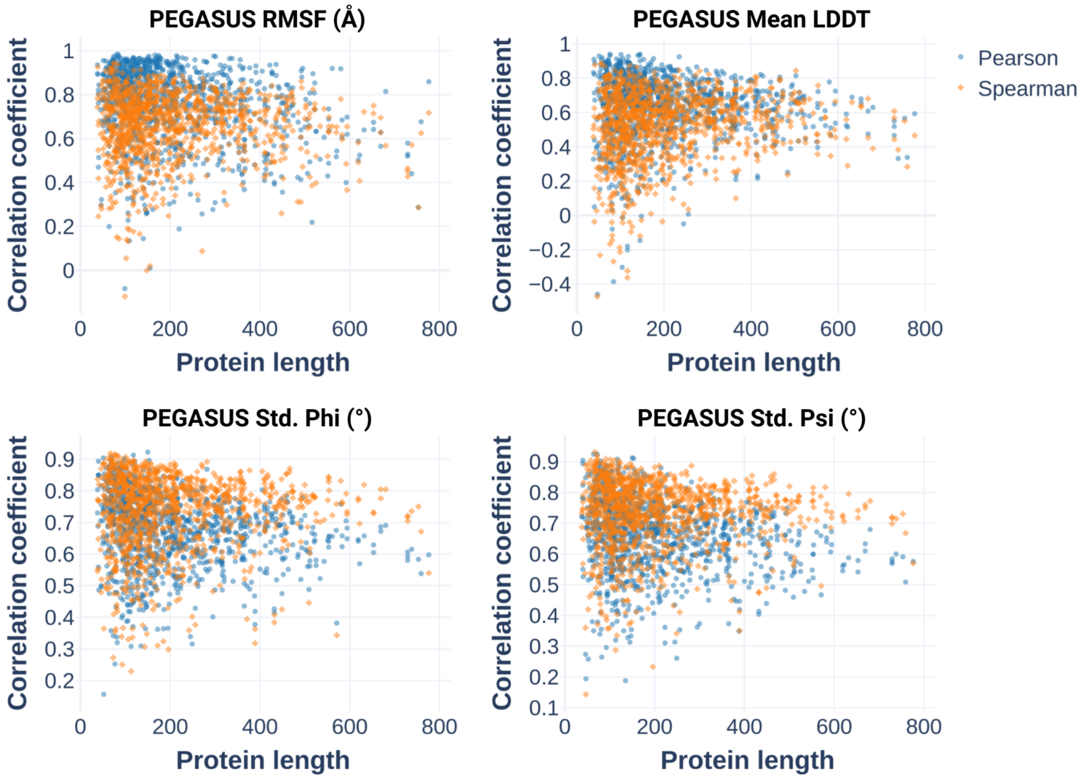

Figure S2: Analysis of PEGASUS prediction accuracy in relation to protein length. Results display 10-fold cross-validation PEGASUS predictions on the ATLAS dataset.

References:

1. Chua,Z.M., Rajesh,A., Sinha,S. and Adams,P.D. (2024) PROTGOAT : Improved automated protein function predictions using Protein Language Models.

2. Elnaggar,A., Essam,H., Salah-Eldin,W., Moustafa,W., Elkerdawy,M., Rochereau,C. and Rost,B. (2023) Ankh: Optimized Protein Language Model Unlocks General-Purpose Modelling.

3. Lin,Z., Akin,H., Rao,R., Hie,B., Zhu,Z., Lu,W., Smetanin,N., Verkuil,R., Kabeli,O., Shmueli,Y., et al. (2023) Evolutionary-scale prediction of atomic-level protein structure with a language model. Science, 379, 1123–1130.

4. Elnaggar,A., Heinzinger,M., Dallago,C., Rehawi,G., Wang,Y., Jones,L., Gibbs,T., Feher,T., Angerer,C., Steinegger,M., et al. (2022) ProtTrans: Toward Understanding the Language of Life Through Self-Supervised Learning. IEEE Trans Pattern Anal Mach Intell, 44, 7112–7127.

Final model architecture and number of parameters

Table S7: Summary of the architectures and training hyperparameters of all final models used for prediction (16 for PEGASUS and 8 for B-factor classification). All networks were trained using early-stopping to avoid overfitting.

Metric	Embedding	Architecture	Batch size	Learning rate	Trainable parameters
RMSF & Mean LDDT	Ankh Large	CONV1 BATCHNORM1 TANH DROPOUT CONV2 BATCHNORM2 TANH CONV3	16	5e-5	2,959,809
	Ankh Base				1,485,249
	ESM36				4,925,889
	ProtT5				1,976,769
Std. Phi & Std. Psi	Ankh Large	CONV1 BATCHNORM1 TANH DROPOUT CONV2	8	1e-5	1,769,985
	Ankh Base				885,249
	ESM t36				2,949,633
	ProtT5				1,180,161
B-factor Strict & B-factor Non-Strict	Ankh Large	CONV1 BATCHNORM1 RELU DROPOUT CONV2 SIGMOID	128	1e-5	5,310,338
	Ankh Base				1,328,066
	ESM36				14,748,802
	ProtT5				2,360,578

Correlation of PEGASUS metrics with AlphaFold 2 pLDDT

Interestingly, PEGASUS slightly outperforms AlphaFold 2's pLDDT in terms of Pearson correlation, achieving 0.75 compared to 0.73, all at a fraction of the computational cost. However, it falls behind in Spearman correlation, with values of 0.66 versus AlphaFold 2's 0.70 (see Fig. S4).

Furthermore, we observe that AlphaFold 2's pLDDT exhibits limited correlation with the actual standard deviations of Phi and Psi angles (Std. Phi & Std. Psi) obtained from molecular dynamics simulations. In contrast, PEGASUS's predictions show stronger correlations with these values, useful for analysing very local motions within proteins. Once again, ESMFold is outperformed by the other methods.

Table S8: Comparison of the performance of PEGASUS, AlphaFold 2 and ESMFold in representing the different flexibility metrics predicted by PEGASUS, using 10-fold cross-validation on the ATLAS dataset.

	Pearson correlation			Spearman correlation		
Tool	PEGASUS predictions	AlphaFold 2 pLDDT	ESMFold pLDDT	PEGASUS predictions	AlphaFold 2 pLDDT	ESMFold pLDDT
RMSF	**0.75 ± 0.02**	0.73 ± 0.01	0.58 ± 0.03	0.66 ± 0.02	**0.70 ± 0.01**	0.56 ±0.03
Std. Phi	**0.68 ± 0.01**	0.35 ± 0.01	0.37 ± 0.02	**0.73 ± 0.01**	0.44 ± 0.02	0.40 ± 0.02
Std. Psi	**0.66 ± 0.02**	0.45 ± 0.01	0.42 ± 0.02	**0.73 ± 0.01**	0.50 ± 0.01	0.43 ± 0.02
Mean LDDT	**0.63 ± 0.02**	**0.63 ± 0.02**	0.58 ± 0.02	0.52 ± 0.03	**0.61 ± 0.02**	0.55 ± 0.03

PEGASUS point mutation prediction

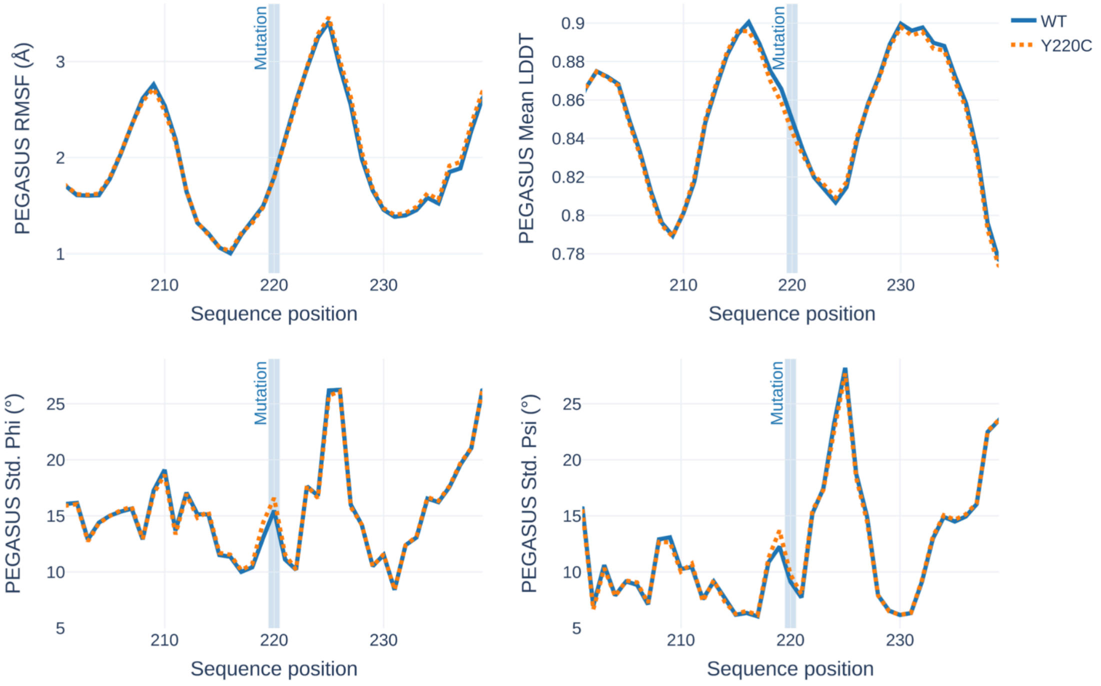

Figure S3: Evaluation of Y220C mutation impact on the p53 flexibility. RMSF, Mean LDDT, Std. Phi and Std. Psi are predicted using PEGASUS, for both wild type and Y220C mutation. Mutation zone is highlighted in light blue.

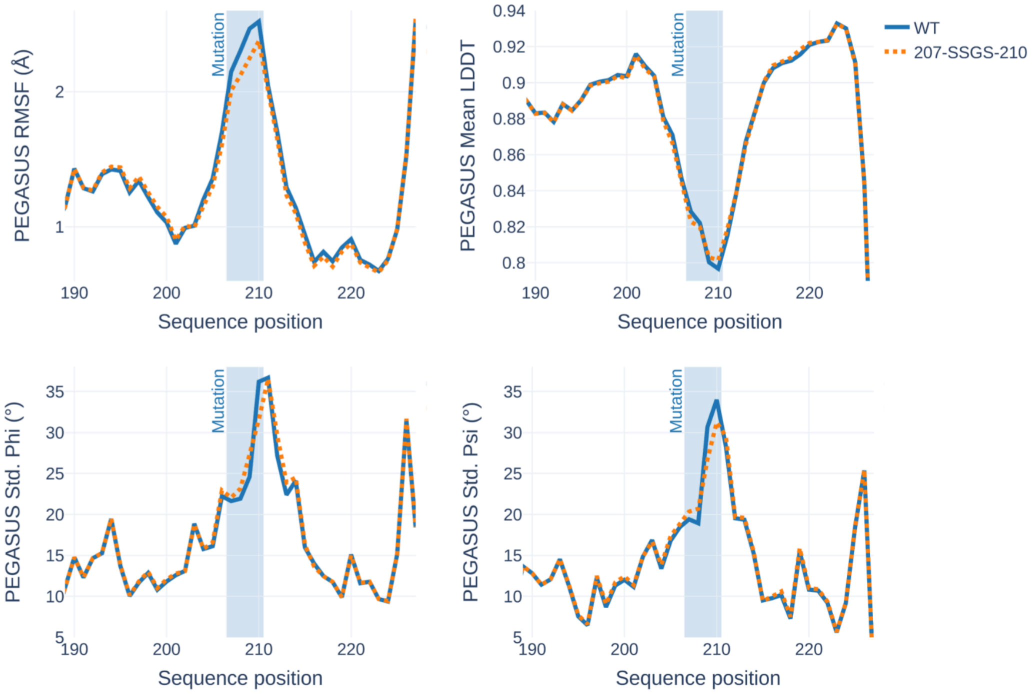

Figure S4: Evaluation of 207-SSGS-210 mutation impact on the GH11 xylanase XynCDBFV flexibility. RMSF, Mean LDDT, Std. Phi and Std. Psi are predicted using PEGASUS, for both wild type and 207-SSGS-210 mutation. Mutation zone is highlighted in light blue.

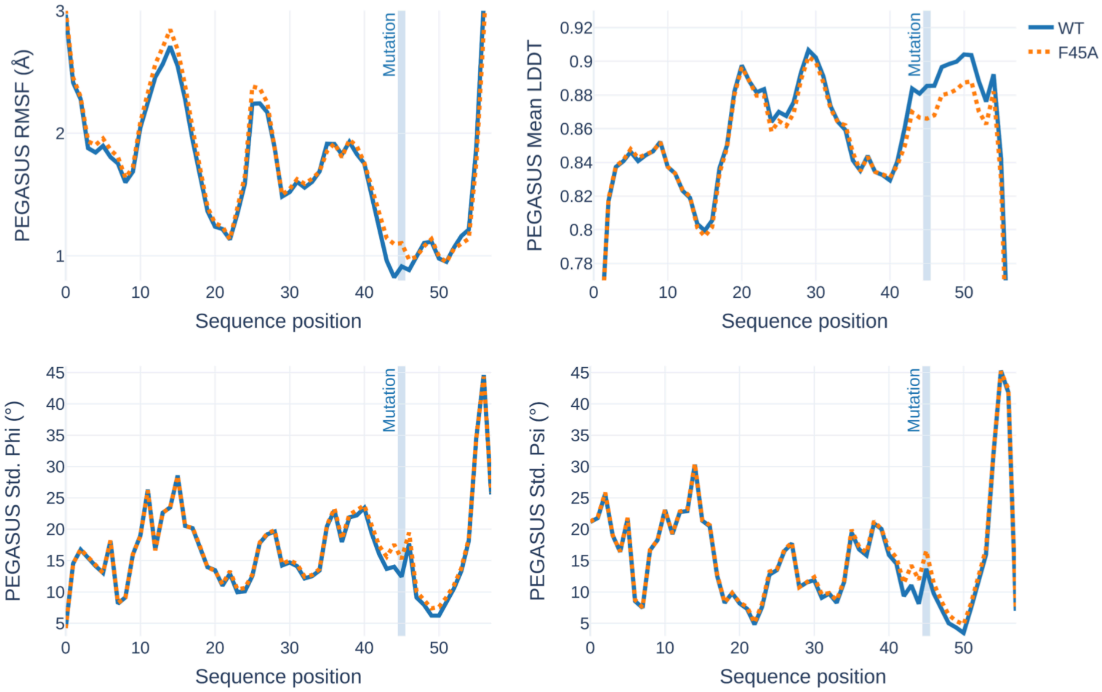

Figure S5: Evaluation of F45A mutation impact on the bovine pancreatic trypsin inhibitor flexibility. RMSF, Mean LDDT, Std. Phi and Std. Psi are predicted using PEGASUS, for both wild type and F45A mutation. Mutation zone is highlighted in light blue.

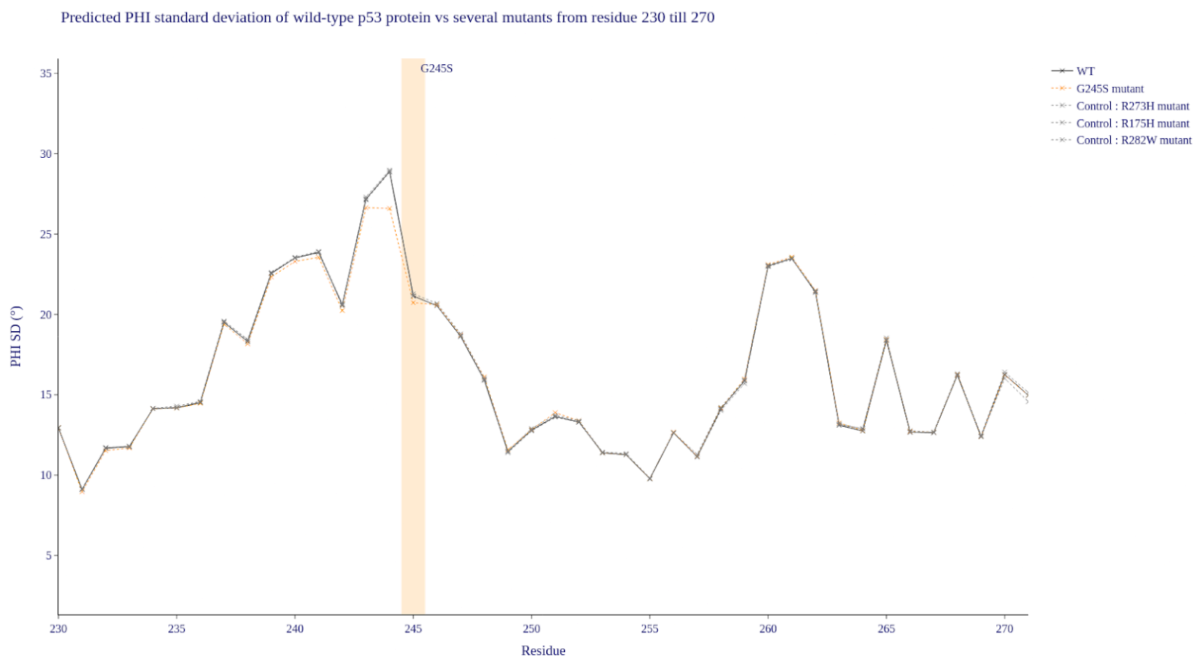

Figure S6: Evaluation of G245S mutation impact on the p53 flexibility for RMSF. To highlight that this impact is due to mutational effect rather than any artefactual model error, the flexibility profiles of destabilising point mutants R273H, R175H and R282W have also been plotted. All these are known to have a locally increased flexibility at the residues of mutation ([Li et al 2020](#)), so are not expected to vary from the flexibility profile of wildtype p53 between residues 230 and 270.

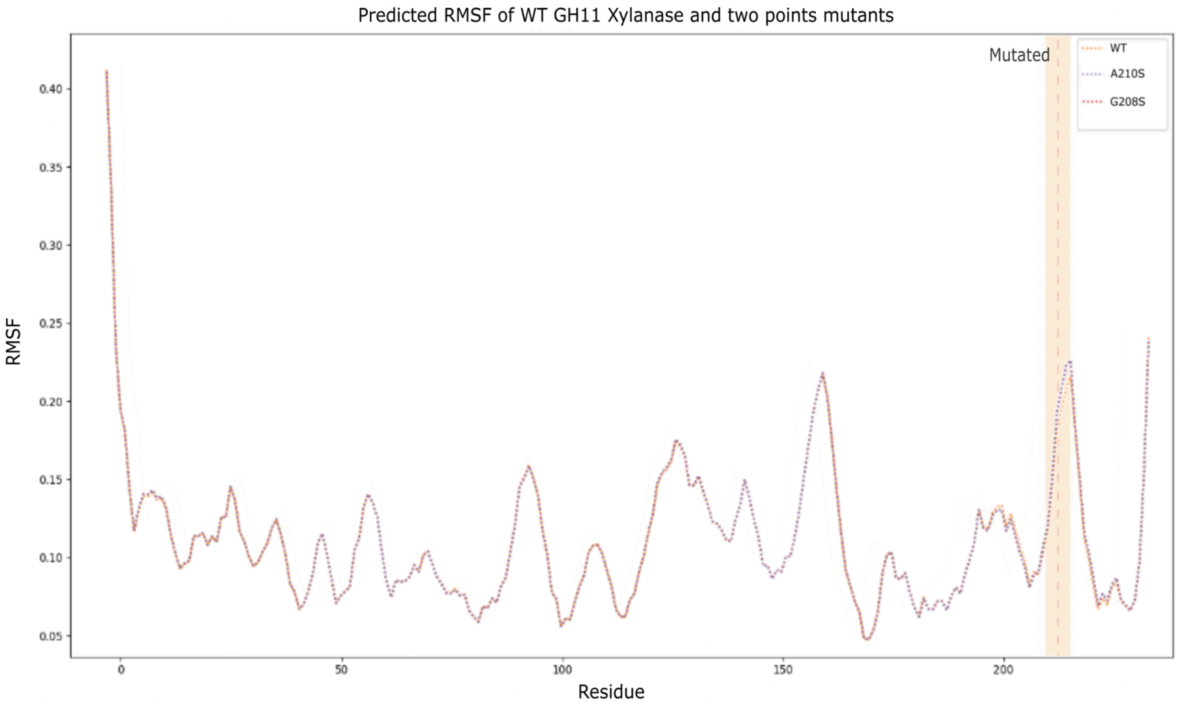

Figure S7: Evaluation of A210S and G208S mutations impact on the RMSF of GH11 Xylanase.

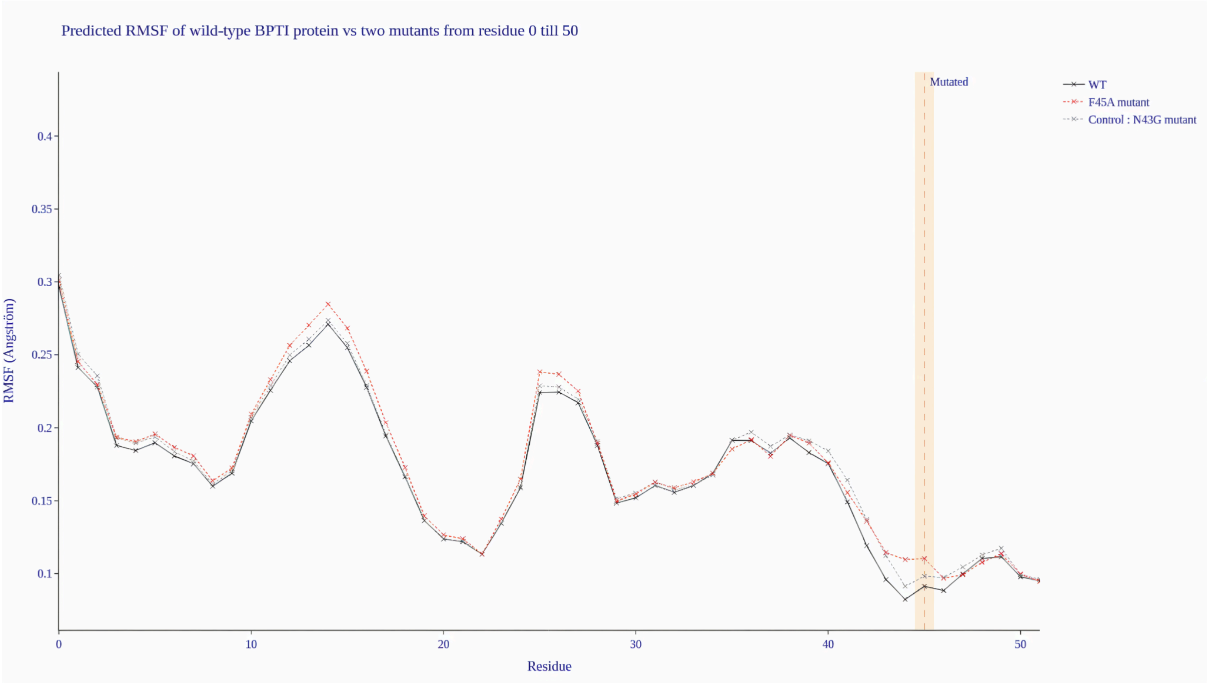

Figure S8: Evaluation of F45A and N43G mutations impact on the bovine pancreatic trypsin inhibitor flexibility for RMSF.

Web server implementation

The PEGASUS web server, available at https://www.dsimb.inserm.fr/PEGASUS, allows users to submit up to 100 protein sequences by job, in FASTA format by either pasting the sequences directly or uploading a file. For larger or more time-consuming jobs, users are encouraged to provide an email address to receive notifications when predictions are complete, or install the local Docker image version. Upon submission, the server performs a validation check to ensure that all sequences contain only standard residues and are no longer than 1000 amino acids. After validation, users are redirected to a progress page that displays the different stages of the job and provides an estimated time for completion. First, the sequences are encoded by four protein language models (pLMs) using a CPU compute node optimised for efficient processing. Next, the PEGASUS models are loaded, and the predictions for each metric are averaged across the pLM predictors. Finally, a dedicated results page is generated for each sequence, accessible through a summary page.

Each results page begins with a brief summary of job parameters, followed by a general flexibility profile displayed using RCSB PDB Saguaro 1D Feature Viewer [1], which highlights the main flexibility features. A more detailed report, featuring interactive Plotly graphs (https://plotly.com/javascript), allows users to zoom into regions of interest and view standard deviations between the predictions from the four embeddings. All predictions are also available for download in tab-separated value format for further analysis.

The server interface is built using HTML and CSS, with the Bootstrap 5 framework (https://getbootstrap.com) for responsive design, and JavaScript/jQuery for interactivity. The back-end is powered by Python and Bash scripts.

PEGASUS predictions for individual sequences are almost instantaneous. For maximal size of allowed job, 100 sequences of 1000 residues each, computational time is of about 20 minutes. The more detailed information on expected calculation time in function of the total number of residues in batch of sequences is provided in Figure S1. Waiting time can be impacted by the server load by other users.

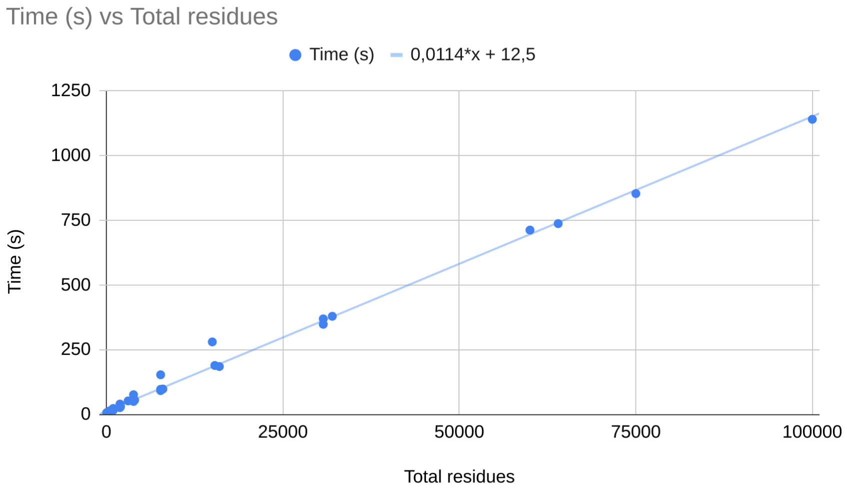

Figure S9. PEGASUS web-server computational time in function of the total number of protein residues in the submitted batch of protein sequences.

References:

1. Segura,J., Rose,Y., Westbrook,J., Burley,S.K. and Duarte,J.M. (2021) RCSB Protein Data Bank 1D tools and services. Bioinforma. Oxf. Engl., 36, 5526–5527.

Performance evaluation for different types of amino acids: ATLAS dataset

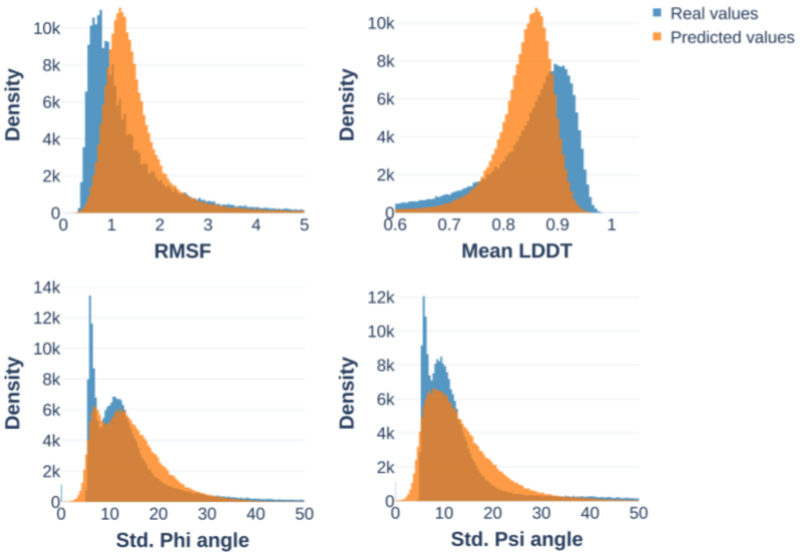

Figure S10: Distributions of experimental and predicted flexibility measures.
For visualization purposes, the distributions are capped: RMSF values originally reaching up to 25 Å, standard deviations of Phi and Psi angles up to 130°, and Mean LDDT values limited to a minimum of 0.

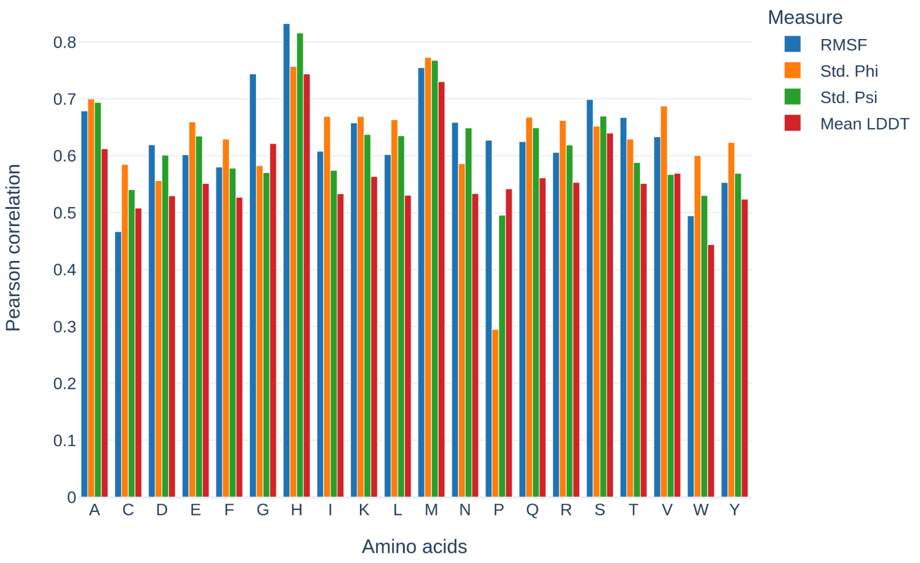

Figure S11: Residue-wise Pearson correlation coefficients between PEGASUS prediction and MD values, for each flexibility measurement.

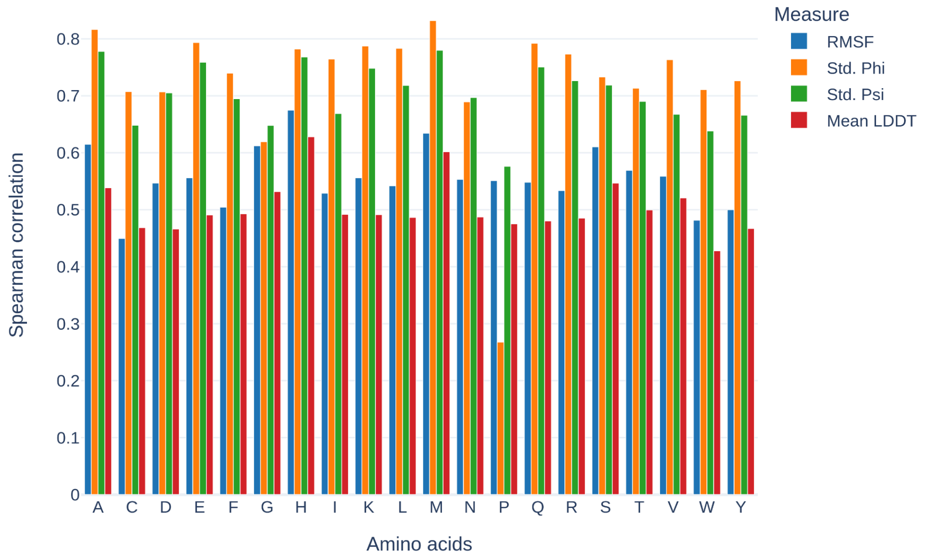

Figure S12: Residue-wise Spearman correlation coefficients between PEGASUS prediction and MD values, for each flexibility measurement.

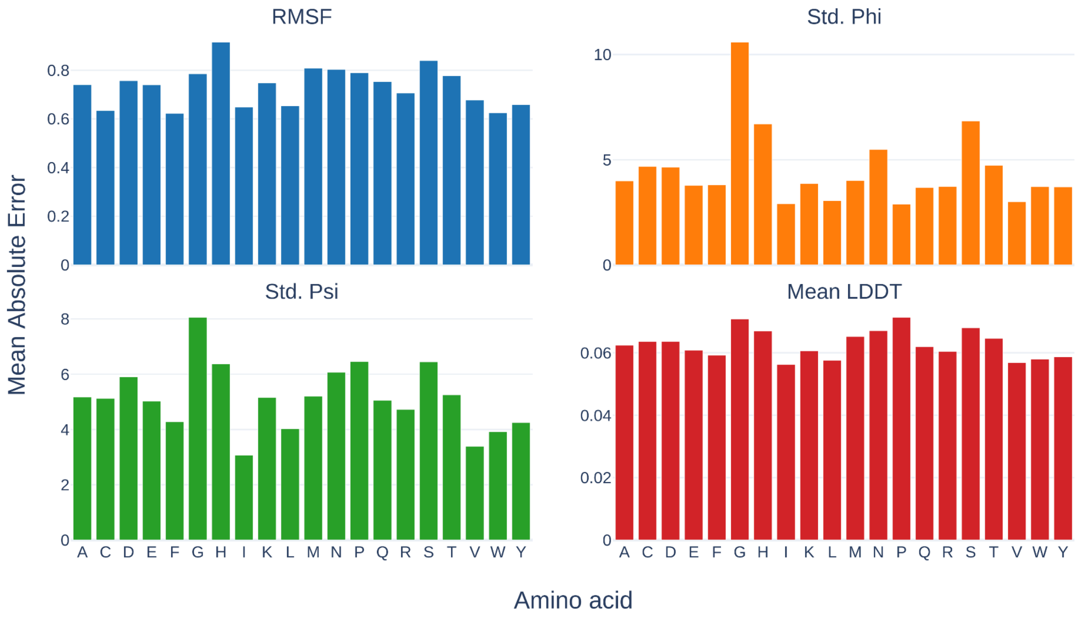

Figure S13: Residu-wise MAE between PEGASUS prediction and MD values, for each flexibility measurement.

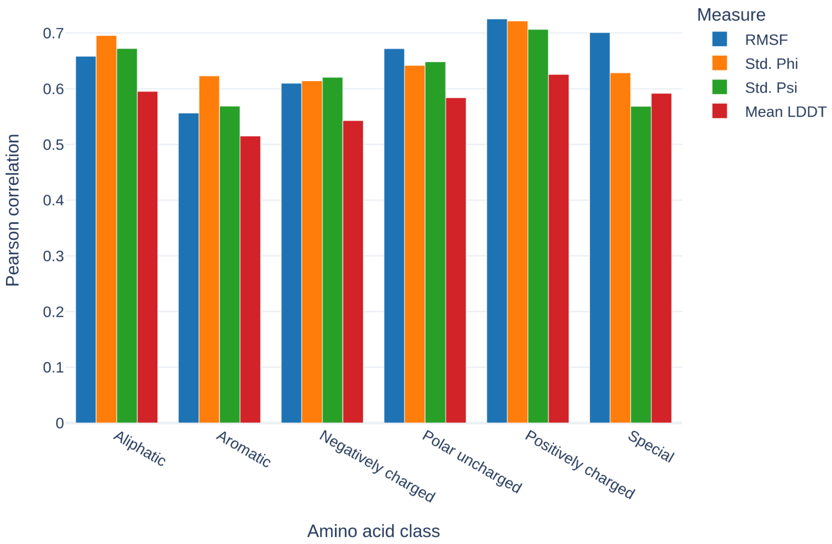

Figure S14: Pearson correlation coefficients between PEGASUS prediction and MD values for different amino acid classes, for each flexibility measurement.

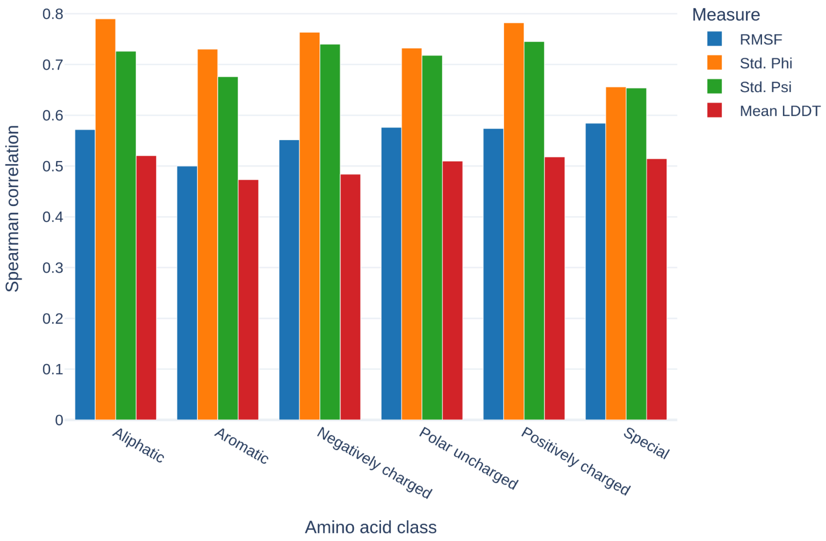

Figure S15: Spearman correlation coefficients between PEGASUS prediction and MD values for different amino acid classes, for each flexibility measurement.

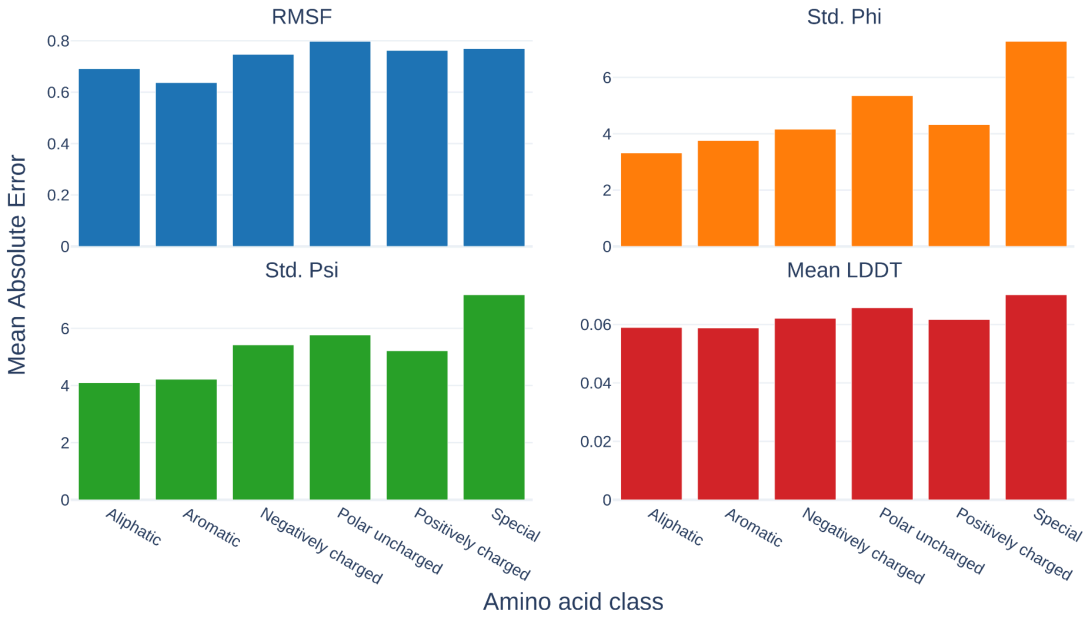

Figure S16: MAE between PEGASUS prediction and MD values for different amino acid classes, for each flexibility measurement.

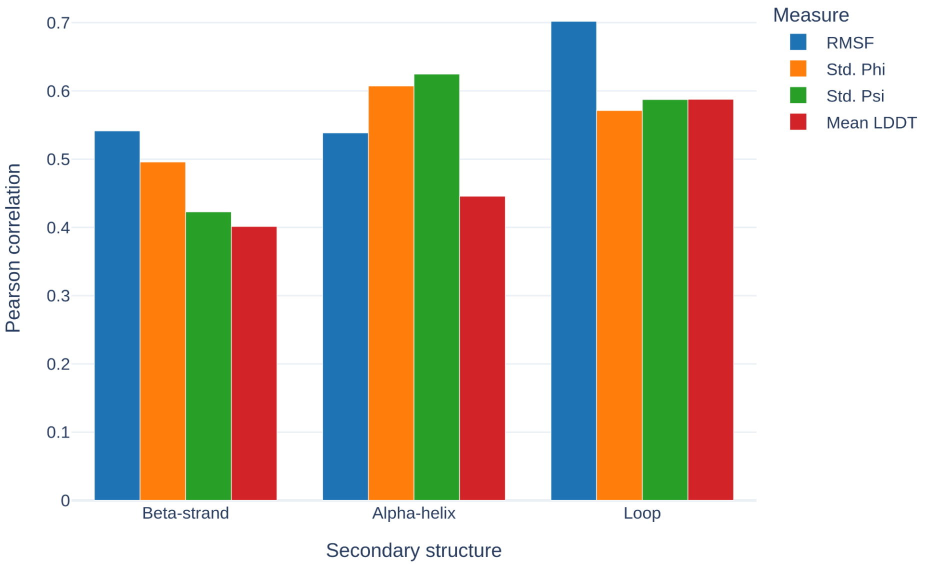

Figure S17: Pearson correlation coefficients between PEGASUS prediction and MD values for different DSSP secondary structure classes, for each flexibility measurement.

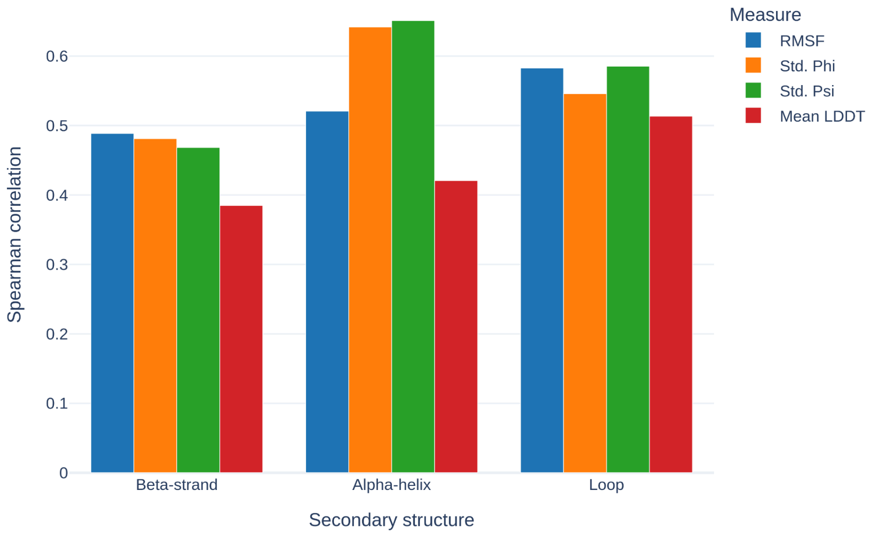

Figure S18: Spearman correlation coefficients between PEGASUS prediction and MD values for different DSSP secondary structure classes, for each flexibility measurement.

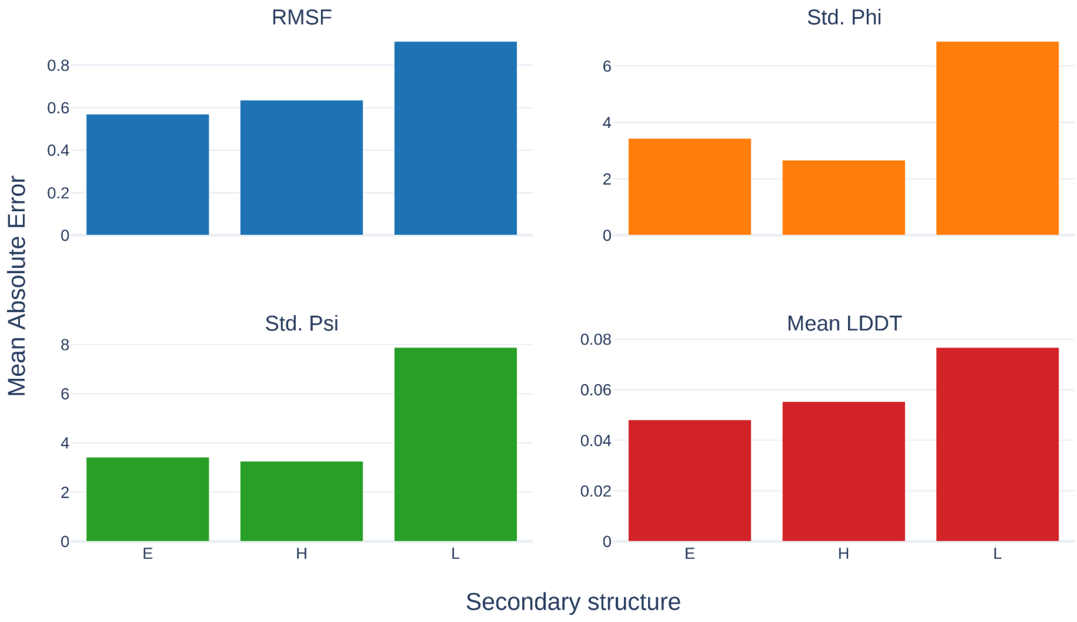

Figure S19: MAE between PEGASUS prediction and MD values for different DSSP secondary structure classes, for each flexibility measurement.

Comparison with degree of protein disorder for different amino acid types: CheZOD dataset

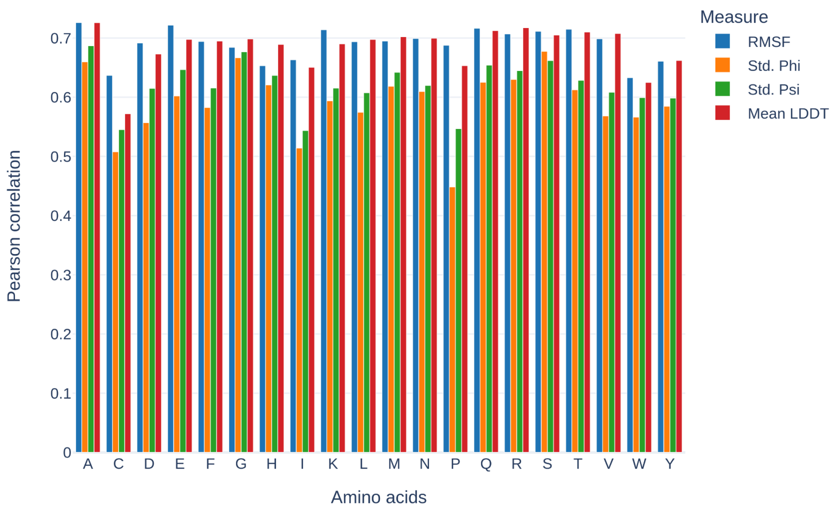

Figure S20: Pearson correlation coefficients between PEGASUS prediction and MD values for different amino acid types, for each flexibility measurement calculated for CheZOD dataset.

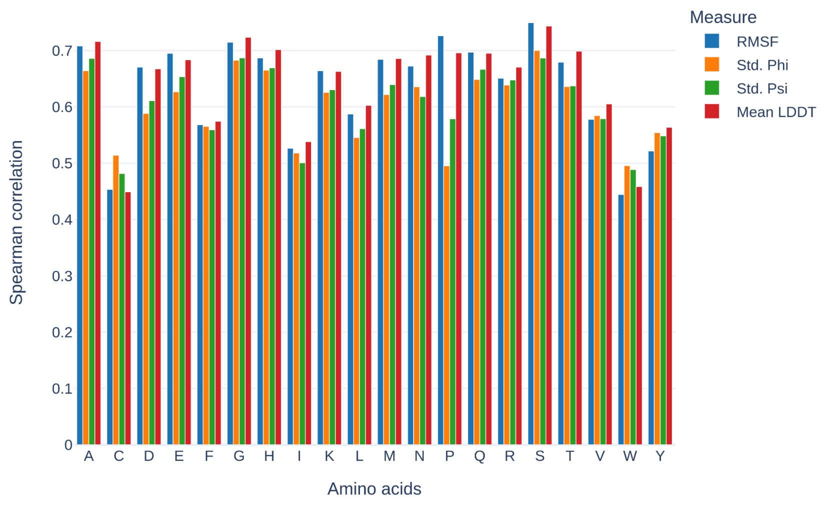

Figure S21: Spearman correlation coefficients between PEGASUS prediction and MD values for different amino acid types, for each flexibility measurement calculated for CheZOD dataset.

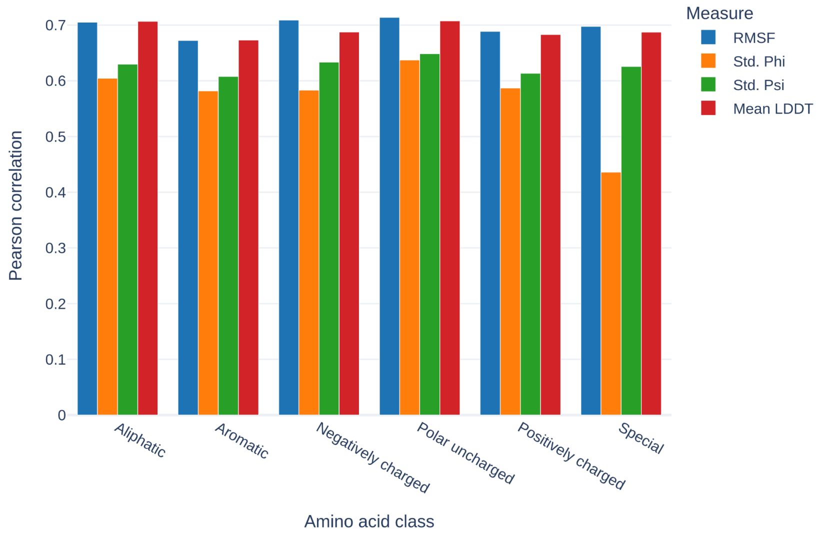

Figure S22: Pearson correlation coefficients between PEGASUS prediction and MD values for different amino acid classes, for each flexibility measurement calculated for CheZOD dataset.

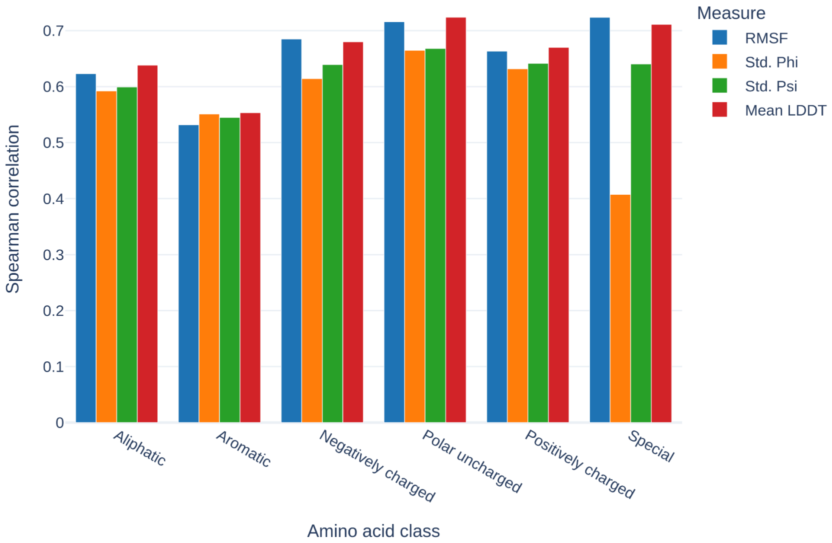

Figure S23: Spearman correlation coefficients between PEGASUS prediction and MD values for different amino acid classes, for each flexibility measurement calculated for CheZOD dataset.

Top-Ranked Proteins by Correlation and Error Metrics

RMSF plots

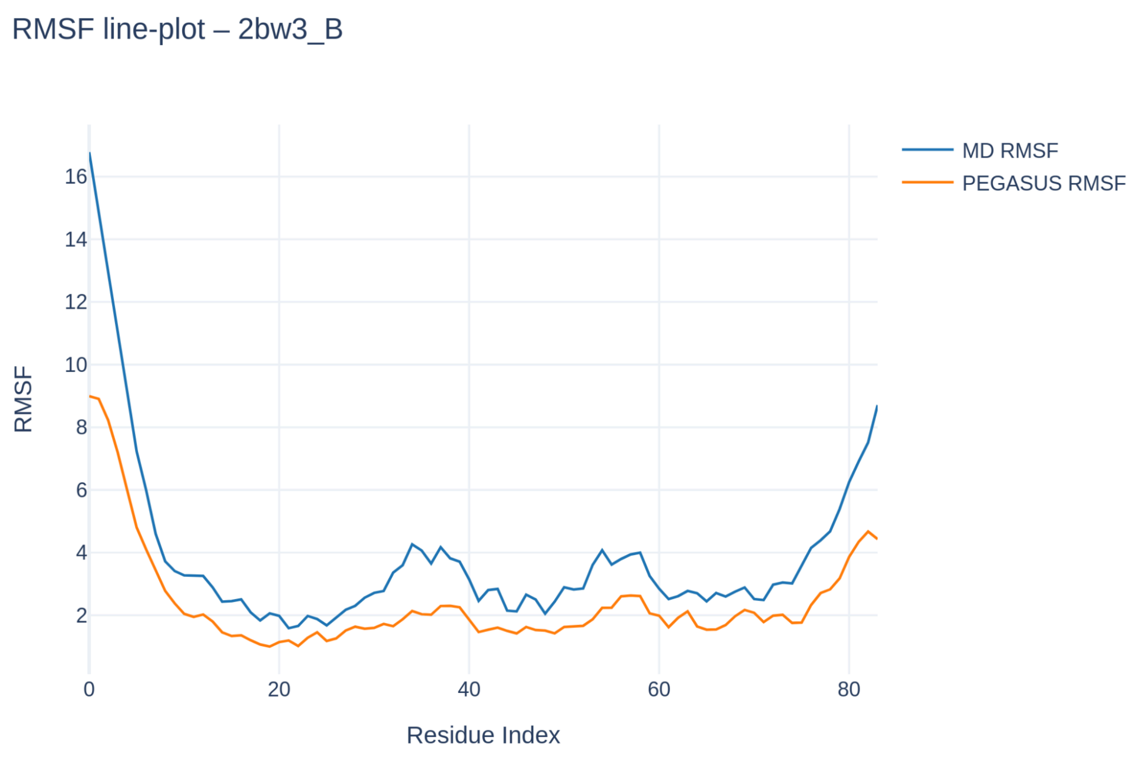

Figure S24: Line-plot of RMSF from PEGASUS and MD for the protein with the strongest Pearson correlation.

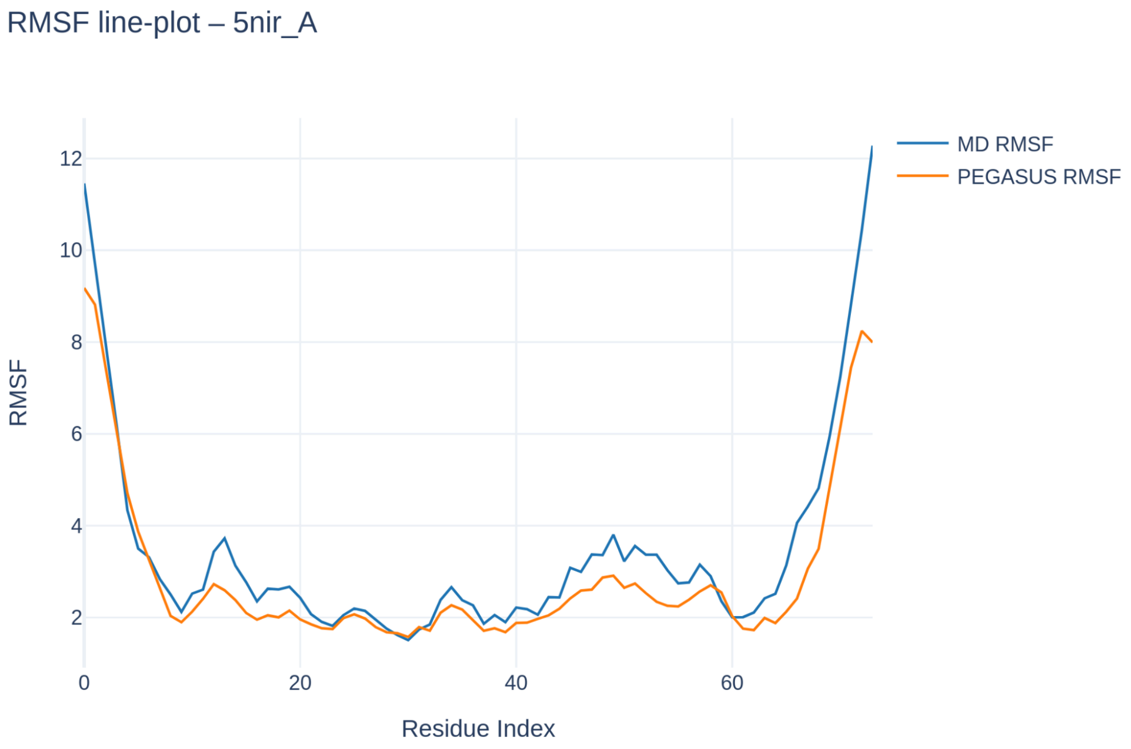

Figure S25: Line-plot of RMSF from PEGASUS and MD for the protein with the strongest Spearman correlation.

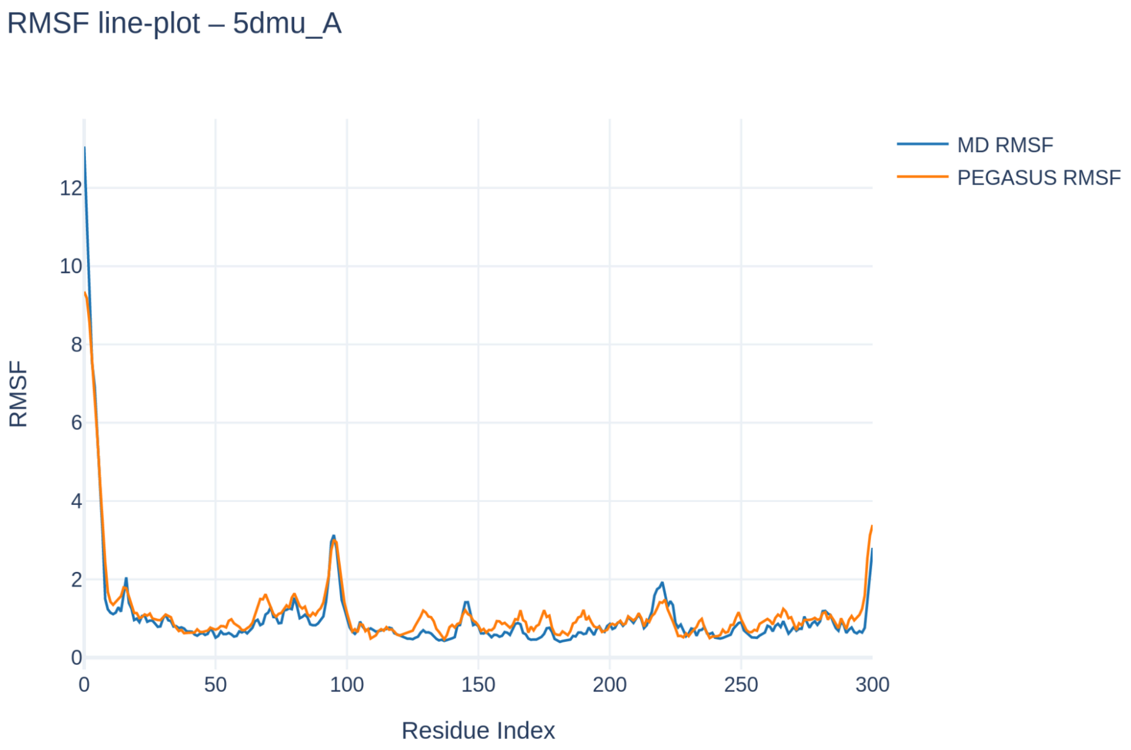

Figure S26: Line-plot of RMSF from PEGASUS and MD for the protein with the lowest MAE.

Std. Phi plots

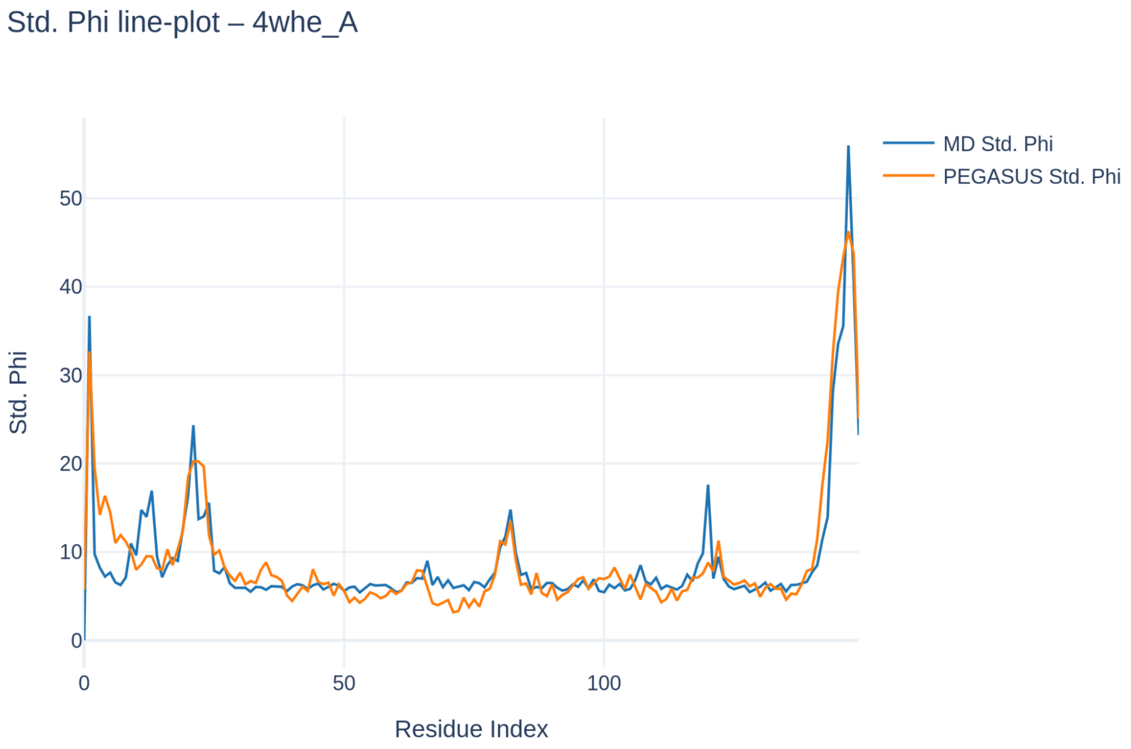

Figure S27: Line-plot of Std. Phi from PEGASUS and MD for the protein with the strongest Pearson correlation & the lowest MAE.

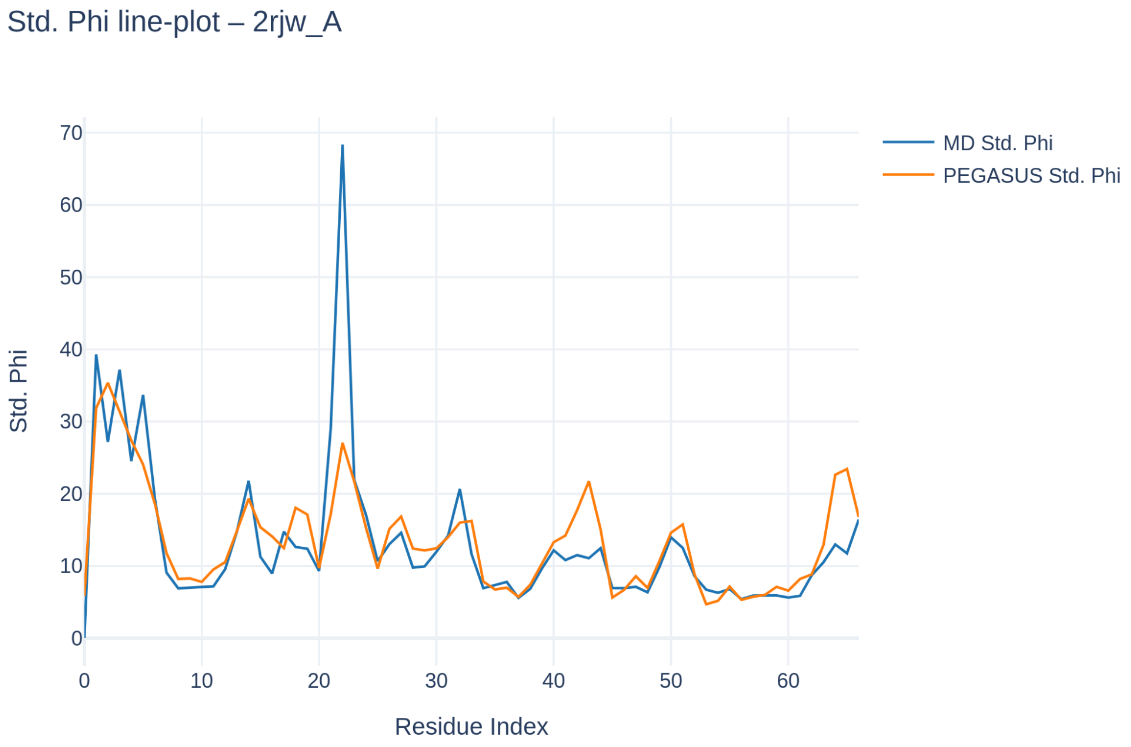

Figure S28: Line-plot of Std. Phi from PEGASUS and MD for the protein with the strongest Spearman correlation.

Std. Psi plots

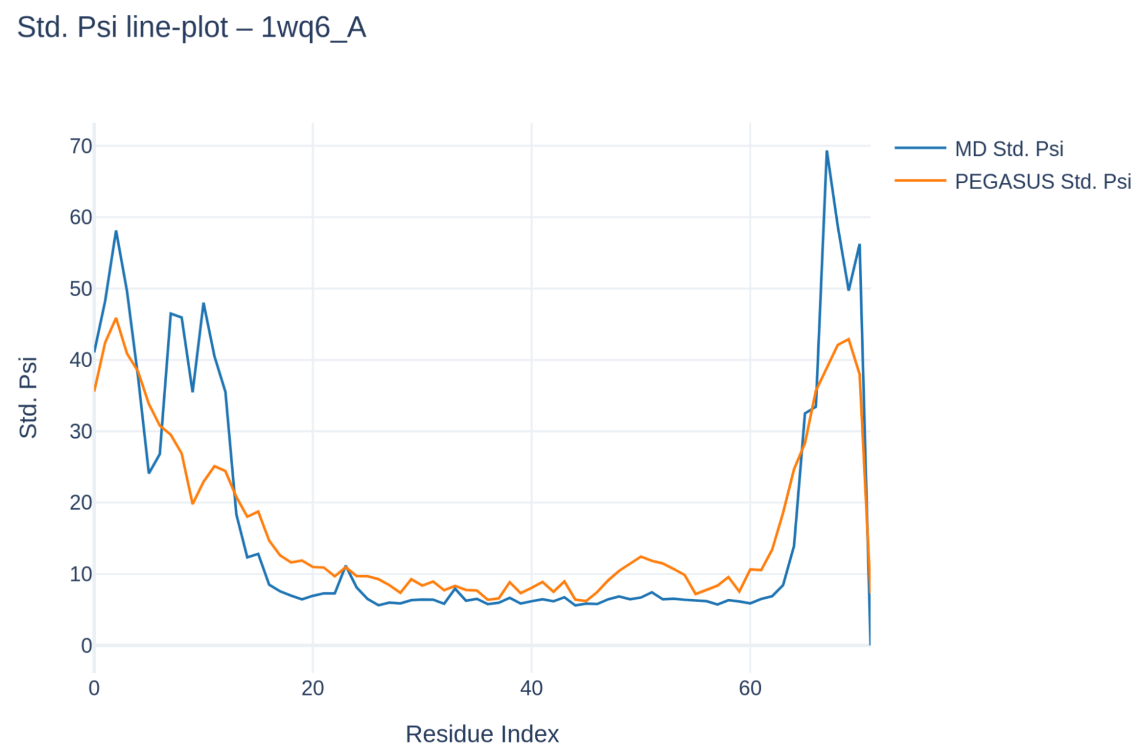

Figure S29: Line-plot of Std. Psi from PEGASUS and MD for the protein with the strongest Pearson correlation.

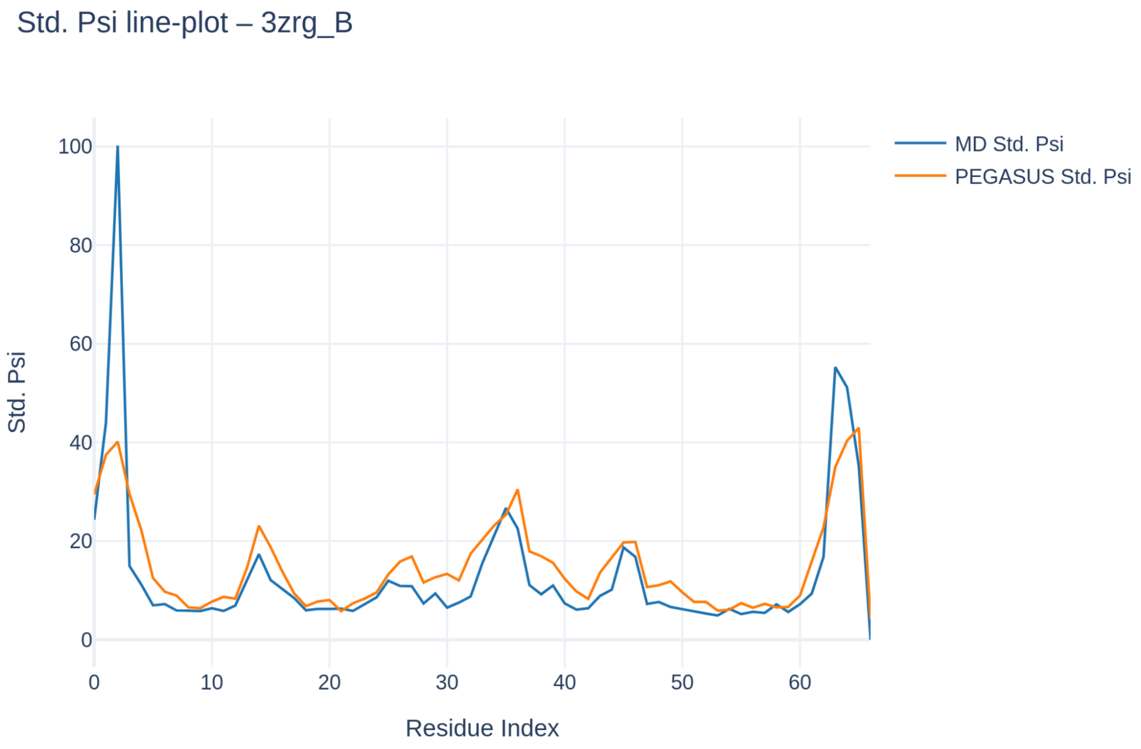

Figure S30: Line-plot of Std. Psi from PEGASUS and MD for the protein with the strongest Spearman correlation.

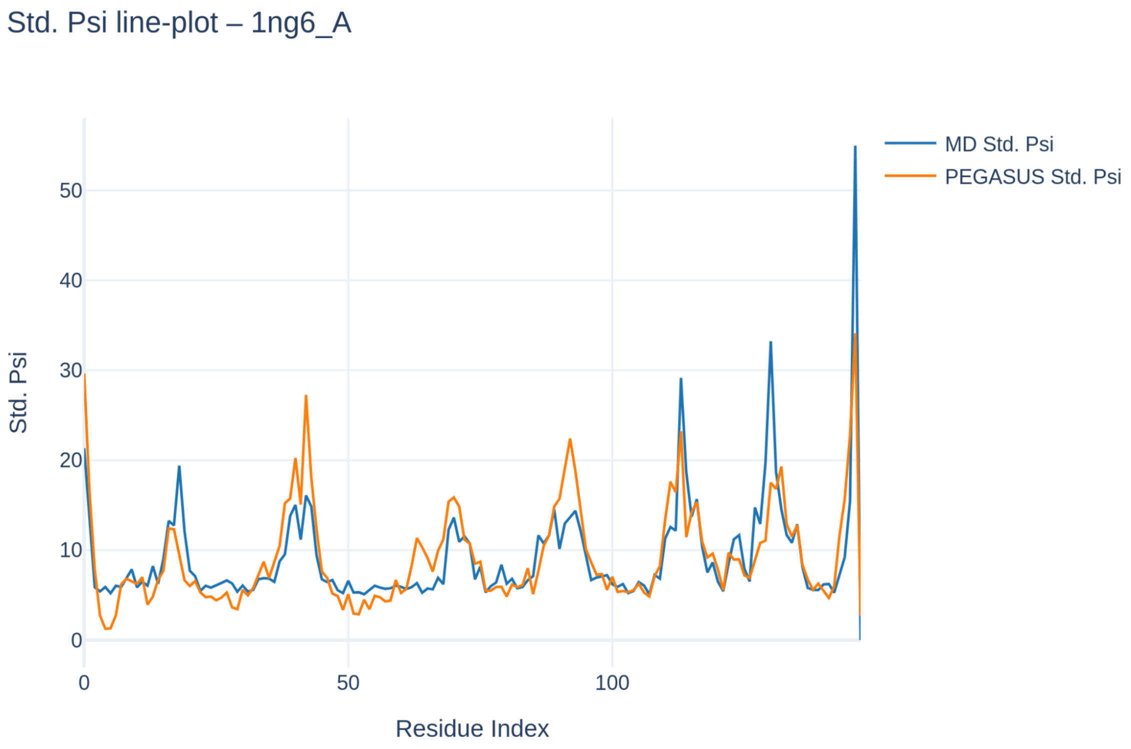

Figure S31: Line-plot of Std. Psi from PEGASUS and MD for the protein with the lowest MAE.

Mean LDDT plots

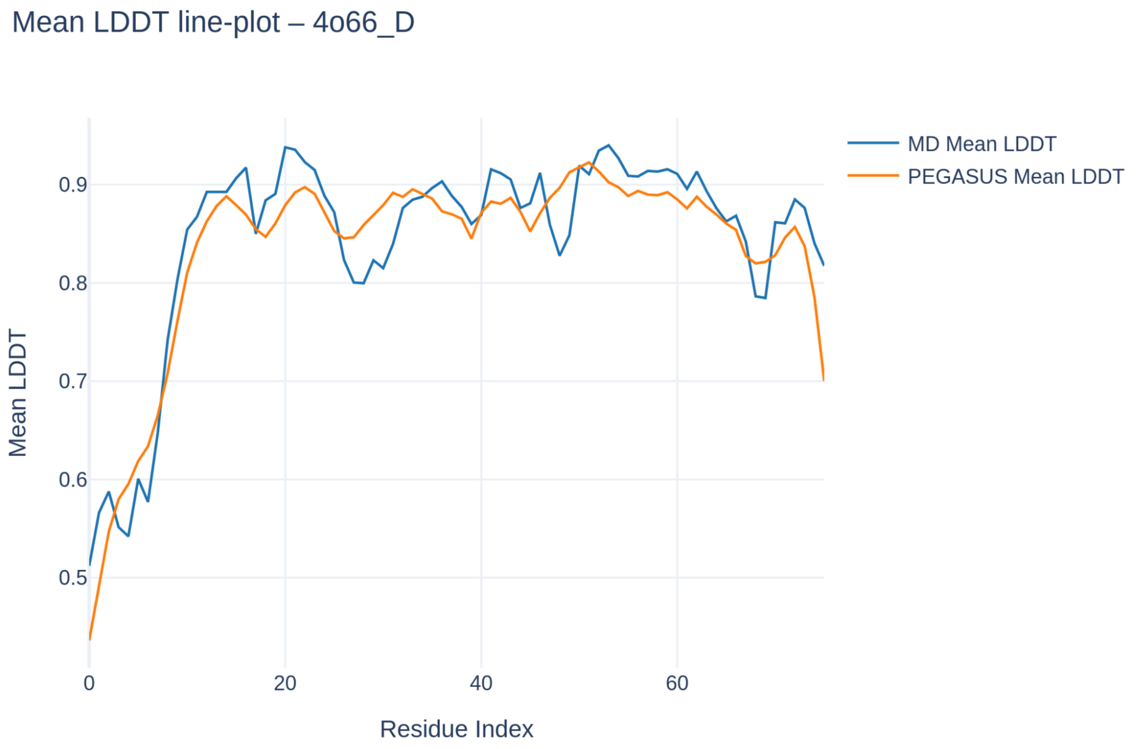

Figure S32: Line-plot of Mean LDDT from PEGASUS and MD for the protein with the strongest Pearson correlation.

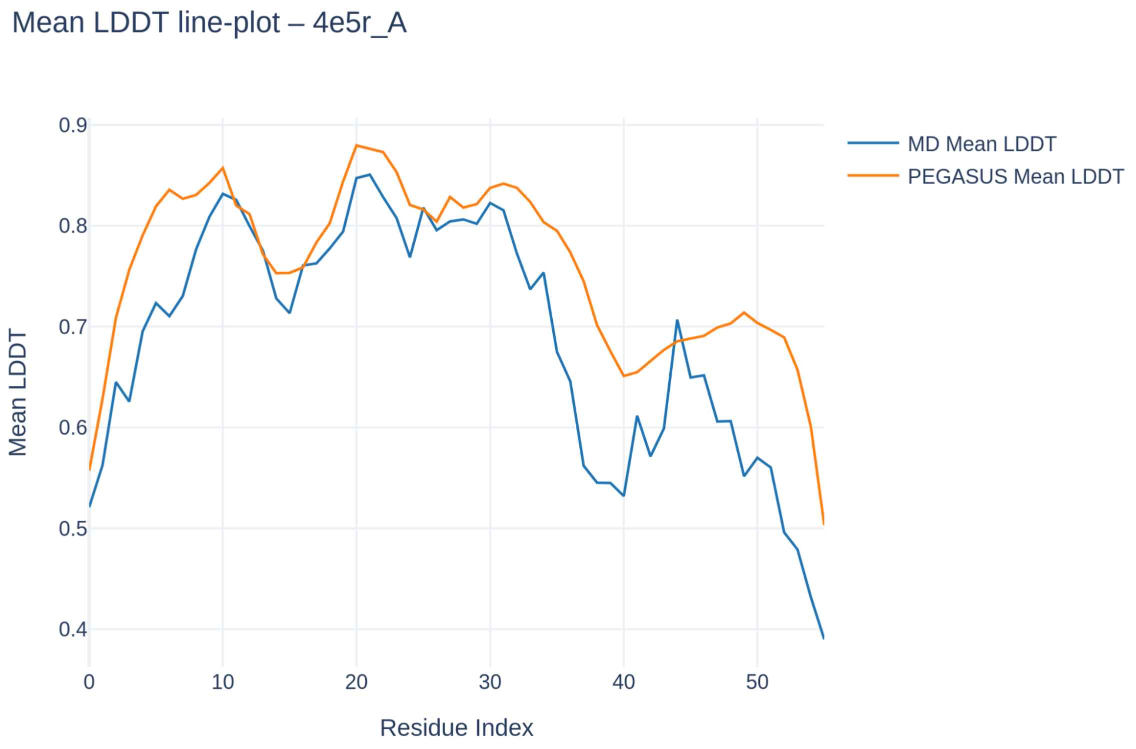

Figure S33: Line-plot of Mean LDDT from PEGASUS and MD for the protein with the strongest Spearman correlation.

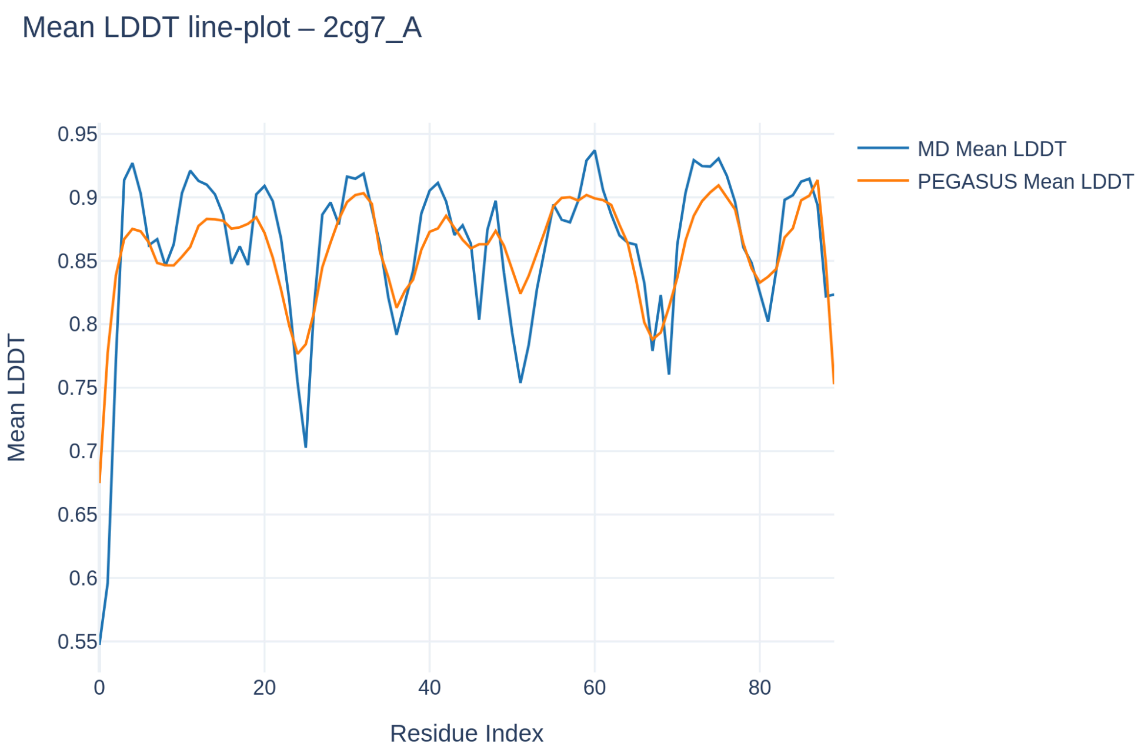

Figure S34: Line-plot of Mean LDDT from PEGASUS and MD for the protein with the lowest MAE.
